# Hidden Hearing Loss? No Effect of Common Recreational Noise Exposure on Cochlear Nerve Response Amplitude in Humans

**DOI:** 10.3389/fnins.2017.00465

**Published:** 2017-09-01

**Authors:** Sarah K. Grinn, Kathryn B. Wiseman, Jason A. Baker, Colleen G. Le Prell

**Affiliations:** ^1^School of Behavioral and Brain Sciences, University of Texas at Dallas Dallas, TX, United States; ^2^College of Public Health and Health Professions, University of Florida Gainesville, FL, United States

**Keywords:** synaptopathy, hidden hearing loss, noise induced hearing loss (NIHL), recreational noise, temporary threshold shift (TTS), speech-in-noise, words in noise (WIN), action potential (AP)

## Abstract

This study tested hypothesized relationships between noise exposure and auditory deficits. Both retrospective assessment of potential associations between noise exposure history and performance on an audiologic test battery and prospective assessment of potential changes in performance after new recreational noise exposure were completed.

**Methods:** 32 participants (13M, 19F) with normal hearing (25-dB HL or better, 0.25–8 kHz) were asked to participate in 3 pre- and post-exposure sessions including: otoscopy, tympanometry, distortion product otoacoustic emissions (DPOAEs) (f2 frequencies 1–8 kHz), pure-tone audiometry (0.25–8 kHz), Words-in-Noise (WIN) test, and electrocochleography (eCochG) measurements at 70, 80, and 90-dB nHL (click and 2–4 kHz tone-bursts). The first session was used to collect baseline data, the second session was collected the day after a loud recreational event, and the third session was collected 1-week later. Of the 32 participants, 26 completed all 3 sessions.

**Results:** The retrospective analysis did not reveal statistically significant relationships between noise exposure history and any auditory deficits. The day after new exposure, there was a statistically significant correlation between noise “dose” and WIN performance overall, and within the 4-dB signal-to-babble ratio. In contrast, there were no statistically significant correlations between noise dose and changes in threshold, DPOAE amplitude, or AP amplitude the day after new noise exposure. Additional analyses revealed a statistically significant relationship between TTS and DPOAE amplitude at 6 kHz, with temporarily decreased DPOAE amplitude observed with increasing TTS.

**Conclusions:** There was no evidence of auditory deficits as a function of previous noise exposure history, and no permanent changes in audiometric, electrophysiologic, or functional measures after new recreational noise exposure. There were very few participants with TTS the day after exposure - a test time selected to be consistent with previous animal studies. The largest observed TTS was approximately 20-dB. The observed pattern of small TTS suggests little risk of synaptopathy from common recreational noise exposure, and that we should not expect to observe changes in evoked potentials for this reason. No such changes were observed in this study. These data do not support suggestions that common, recreational noise exposure is likely to result in “hidden hearing loss”.

## Introduction

The mammalian auditory system is susceptible to noise exposure injury resulting from damage to cells in the inner ear. Changes in function can be temporary or permanent (for review, see Ryan et al., 2016). The Occupational Safety and Health Administration (OSHA) federal noise regulations define an auditory “standard threshold shift” as a permanent change in hearing threshold, relative to one's baseline audiogram, of an average of 10-dB or more at 2, 3, and 4 kHz in either ear (OSHA, 1983). A temporary threshold shift (TTS), by definition, does not meet this regulatory standard for a workplace-induced noise injury. However, recent findings suggest that large TTS may result in permanent synaptic loss (Kujawa and Liberman, 2009), followed by slow, progressive neural degeneration (Kujawa and Liberman, 2006). Thus, exposures that result in TTS may be more harmful than previously believed (Kujawa and Liberman, 2015).

Noise exposures that result in a relatively robust TTS 24-h after the noise exposure have been accompanied by loss of the synaptic connections between inner hair cells (IHCs) and the afferent neurons in mice (Kujawa and Liberman, 2009; Wang and Ren, 2012; Fernandez et al., 2015) and guinea pigs (Lin et al., 2011; Furman et al., 2013). With this decrease in the neural output of the cochlea, the amplitude of Wave-I of the sound-evoked auditory brainstem response (ABR) is permanently reduced, even though the ABR Wave-I threshold remains unchanged (for reviews, see Kujawa and Liberman, 2015; Liberman and Kujawa, 2017). Because these noise exposures result in damage that cannot be detected by conventional audiometric threshold assessment, this synaptopathic injury has been referred to as “hidden hearing loss”, a term originally coined by Schaette and McAlpine (2011). Synaptopathic injury appears to be biased toward low spontaneous firing rate neurons, which have higher response thresholds and are responsible for coding higher intensity (suprathreshold) sounds (Furman et al., 2013). In contrast, synaptic contacts with the high-spontaneous rate neurons, which have lower response thresholds and are responsible for coding lower intensity sounds (i.e., audiometric thresholds), appear to be largely unaffected. This may explain why the threshold audiogram is not sensitive to loss of IHCs (Lobarinas et al., 2013) or afferent synapses (Kujawa and Liberman, 2009).

It has been suggested that noise-induced neuropathic damage may explain the disproportionate difficulties some individuals experience processing speech in noisy environments, despite clinically normal hearing thresholds (Kujawa and Liberman, 2009; Lin et al., 2011; Makary et al., 2011). More recently, there have been several suggestions that recreational noise could induce cochlear synaptopathy manifested as difficulty understanding speech in background noise with deficits “hidden” behind a standard audiogram. Liberman (2015) points to “the loud pop of Fourth of July fireworks or the roar of the crowds at a football game” as not only affecting hair cells, but also damaging the auditory neurons, and suggests that their research finding “raises questions about the risks of routine exposure to loud music at concerts and clubs and via personal listening devices.” Jensen et al. (2015) similarly point to the increasing sales of portable listening devices, and suggest that there has been a corresponding “shift of ‘at-risk’ users from adults to adolescents.” Suggestions such as these have led multiple groups to seek evidence that would suggest a potential synaptopathic injury in otherwise normal-hearing young adult cohorts (Stamper and Johnson, 2015a,b; Prendergast et al., 2017; Spankovich et al., 2017; Fulbright et al., in press).

Although Stamper and Johnson (2015a) presented evidence that was interpreted as consistent with a synaptopathic noise injury (reduction in ABR Wave-I amplitude) secondary to recreational noise history in normal-hearing young adults, the investigation did not account for differences in ABR Wave-I amplitude as a function of sex. After controlling for sex, the observed effects were limited to females (Stamper and Johnson, 2015b). More recently, Prendergast et al. (2017), Fulbright et al. (in press), and Spankovich et al. (2017) were unable to provide evidence consistent with noise-induced synaptopathic injury in other young adult populations with varying recreational noise histories. However, failure to detect deficits in ABR Wave-I amplitude in young adults with recreational noise exposure histories is perhaps not that surprising. In animal models, shorter or less intense noise exposures that result in smaller TTS changes do not result in synaptopathic injury, functional deficits, or progressive neuronal loss (Hickox and Liberman, 2014; Fernandez et al., 2015; Jensen et al., 2015; Lobarinas et al., 2017). In studies with rodents, 20–30 dB TTS 24 h post noise generally has not been associated with synaptopathic change, whereas 40–50 dB TTS 24 h post noise clearly has been associated with synaptopathic damage. Thus, typical recreational noise exposures commonly experienced by young adults likely are not sufficient to result in an acute neural pathology. The lack of deficits observed in studies assessing young adults with a history of recreational noise exposure (Prendergast et al., 2017; Spankovich et al., 2017; Fulbright et al., in press) does not preclude the possibility that damage emerges with louder, longer, or more frequently repeated noise exposures, such as firearm exposure (Bramhall et al., 2017), explosions (Remenschneider et al., 2014), and blast exposure in the course of military service (Helfer et al., 2011; Gallun et al., 2012a,b; Saunders et al., 2015). The data from Bramhall et al. (2017) are compelling in showing reduced ABR Wave-I amplitude in civilians and military personnel with high noise exposure, and the data from Liberman et al. (2016) raise important questions about the potential for hazard for musicians. The issue of unknown damage-risk criteria for synaptopathic injury and hidden hearing loss is a challenge not only for public health hearing loss prevention efforts targeting adolescents, but also for the protection of noise-exposed workers (for discussion, see Dobie and Humes, 2017; Murphy and Le Prell, 2017).

The current investigation is the first to describe prospective monitoring of young adults attending loud recreational venues for potential changes in both auditory evoked potentials and functional performance (tone detection and speech-in-noise testing) as a consequence of acute recreational noise exposure. The unique features of this study were (1) collection of data pre- and post-noise exposure, (2) the use of a sound-pressure-level meter smartphone app to document exposure during loud events attended by participants, and (3) the integration of functional word-in-noise tests with evoked potential measures in assessing effects of recreational noise. These data were collected with the specific goal of generating evidence that will provide insight into the potential hazards of individual recreational events, as a function of the accrued noise dose, so that future investigations can more precisely target at-risk populations. In addition to the use of prospective test design, the current investigation adds data on the relationship between hearing-in-noise and noise exposure history. Distortion product otoacoustic emission (DPOAE) amplitude was assessed in order to differentiate potential damage to the outer hair cell (OHC) and IHC populations.

## Methods

This study was approved by the Institutional Review Board at the University of Texas at Dallas. Signed consent forms were obtained from participants prior to study enrollment. Participants were recruited from the University of Texas at Dallas campus in Richardson, Texas and the Callier Center for Communication Disorders in Dallas, Texas. All study procedures were performed using dedicated clinical research equipment located at the Callier Center for Communication Disorders in Dallas, TX. All study procedures were conducted by students in their third or fourth year of training in the Doctor of Audiology program. Participants were allowed to withdraw at any time; they were compensated for each laboratory visit.

Participants included 32 young adults (13 male, 19 female; mean age 23.5 years, range 21–27 years). Participants were asked to self-identify sex; we are not aware of any participants for whom gender identity was different from biological sex. All participants met the study enrollment criteria, including normal otoscopic examination bilaterally (visualization of the tympanic membranes with no apparent abnormalities), normal tympanometric examination bilaterally (Type A with 226 Hz probe tone), and normal hearing (defined as thresholds of 25 dB HL or better from 0.25 to 8 kHz bilaterally).

Participants were invited to attend three test sessions. In order to avoid enticing participants to attend a loud recreational event, the second session was specified as being either (A) the day after attending a loud recreational event of their choice, or (B) a second baseline session during which the participant would be retested to establish retest reliability in the absence of attending a loud event. The third session was completed 1-week later. Although having plans to attend a loud event was not an enrollment criterion, all participants already had plans to attend common “loud” recreational events at the time of study enrollment (concert, *n* = 16; multi-day music festival, *n* = 2; bar with live music, *n* = 3; bar with digital music, *n* = 4; dance event, *n* = 3; movie, *n* = 1). The participants self-identified events as “loud,” and there was no duration requirement; as such, the recreational events varied with respect to type, level, and duration.

Noise levels were estimated using the smartphone app “SPL Graph,” installed on each participant's phone prior to event attendance. Data presented by Grinn et al. (2017) showed this app to be accurate within 2-dB of a class 1 sound level meter (SLM) across 25 used (not-new) iPhones (models 5, 5S, 6, 6S, 6S Plus, and 7) for test signals including steady-state broadband noise (90–110 dBA) and five pop songs (85–105 dBA). To assure that individual participants in this study were able to accurately measure sound levels using this app, the app was installed on participant iPhones and accuracy was verified against a class 1 SLM (Brüel and Kjær, type 2250; calibration verified using a Brüel and Kjær Type 4231 calibrator prior to use). At the baseline test session, participants were taught how to use the app and demonstrated the ability to point their phone microphone at a sound source to capture a measurement. Ten instantaneous sound level measurements (dBA) were captured by each participant at various moments throughout their loud event; the average event sound level was estimated using these 10 instantaneous sound level measurements. Event duration was recorded and reported by the participant. Estimated noise dose per individual participant was calculated using 29 CFR 1910.95 Appendix A (OSHA, 1983) based on the measured levels and the reported duration of attendance.

Taken together, the overall design included 3 test sessions completed as follows: (1) baseline test prior to attending a loud event, (2) retest within 24 h after the loud event, (3) retest 1-week after the loud event. Of the 32 participants enrolled in the study, 26 completed all 3 test sessions and their data are included in both the retrospective and prospective analyses. Two additional participants completed the first two test sessions, but not the final 1-week post noise session. Data from these participants was included in the analyses. Three additional participants completed only the baseline test session and their data are included only in the retrospective analysis, as there were no post-noise data to include for these two participants. One additional participant completed the online surveys but did not complete any test sessions; their survey data were excluded as there were no audiologic data for this participant.

### Retrospective noise survey

Participant demographic information and self-reported retrospective noise exposure history were obtained via online survey using Qualtrics. The online survey was created based on the Noise Exposure Questionnaire (NEQ), which has now been used by a variety of groups to retrospectively assess self-reported exposure to occupational and recreational noise (Megerson, 2010; Stamper and Johnson, 2015a; Spankovich et al., 2017; Fulbright et al., in press). This questionnaire, expanded from a similar survey developed by Neitzel et al. (2004), assesses the self-reported frequency of previous exposures to various noisy activities (e.g., concerts, motorcycles, power tools, firearm use, etc.). From these responses, the total noise exposure within the previous year is calculated (for detailed procedures, see Megerson, 2010; Johnson et al., 2017). In brief, each activity is assigned an Exposure Level (EL) based on measured sound levels in previously reported literature. All hours not “assigned” to a noisy activity are assigned a default value of 60-dBA. For each participant, the total number of annual hours of exposure to each loud activity is divided by the reference duration (the number of hours allowed per year based on typical sound levels). These individual activity-specific doses are then summed to estimate total annual noise dose (Annual Exposure, AE).

From the AE—the total annual accumulated noise dose based on the self-reported activities—the L_Aeq8760_ equivalent noise exposure term is derived. There are 8,760 h in a 1-year period (24 h/day × 365 days/year = 8,760 h); of these, some 2,000 h might be assumed to be spent working at some occupation (8 h/day × 5 days/week × 50 weeks per year = 2,000 h). Thus, the total year over which exposure can accrue is approximately two doublings of the typical occupational window. If using a 3-dB exchange rate and an 85-dB criterion level to set a safe exposure limit (as advocated by NIOSH, 1998), then the allowed exposure over 8,760 h should be approximately 6-dB less than the allowed exposure over the 2,000 work h. Thus, the “safe” exposure over 8,760 h has been derived to be 79 dBA. Therefore, L_Aeq8760_ is calculated using the following equation:

$$\begin{array}{l} {\text{L}}_{\text{Aeq}8760}=\left[10\times \text{log}\left(\text{AE}/100\right)\right]+79 \end{array}$$

### Audiologic testing

At each test session, the following clinical measures were performed bilaterally:

#### Otoscopy

Visual examination of the ear canal and tympanic membrane was conducted to assure normal anatomy and no presence of debris. Normal otoscopic outcomes were defined as visualization of the tympanic membrane with no apparent abnormalities.

#### Tympanometry

Tympanometric measures were used to assess the functional status of the middle ear using a Grason Stadler Instruments TympStar Pro in compliance with *ANSI S3.39* and *IEC 601-1* criteria. Normal middle ear function was defined as Type A 226 Hz tympanograms bilaterally.

#### Distortion product otoacoustic emissions (DPOAEs)

The 2f1-f2 distortion product was elicited with two simultaneously presented “primary” tones (f1 and f2) at an f2/f1 ratio of 1.2, with f2 frequencies of 1, 2, 3, 4, 6, and 8 kHz (f1: 55-dB SPL; f2: 45-dB SPL). These levels were selected based on previous studies showing temporary noise-induced changes in DPOAE amplitude were greater at these levels than when f1 and f2 were presented at higher levels (65/55) or lower levels (45/35) (Le Prell et al., 2012, 2016). Two runs were performed per ear at each test session to assure repeatability. DPOAE measurements were obtained using the Interacoustics Eclipse DPOAE Module in combination with an ER10C microphone-earphone assembly and a disposable foam ear tip.

#### Audiometry

Pure-tone air and bone conduction thresholds were obtained at all 3 test sessions (pre-event baseline, the day after the loud event, and 1-week post-noise) using the Modified Hughson-Westlake procedure for frequencies from 0.25 to 8 kHz, with sound levels decreased by 10-dB after each correct detection and increased by 5-dB after each missed stimulus. All audiometric testing was conducted inside a sound-treated booth, using a GSI Audiostar Pro audiometer. ER3-A insert earphones were used for air conduction audiometry and all speech testing. A GSI Audiostar Pro bone oscillator was used for bone conduction audiometry.

#### Speech recognition threshold (SRT)

As part of the standard clinical battery, speech recognition thresholds (SRT) were obtained using a recorded spondee list from the GSI Audiostar Pro audiometer. The spondee words have two syllables which are pronounced with equal emphasis (e.g., “toothbrush”). The SRT is the minimum signal level at which the listener can correctly identify 50% of the speech material presented (Plomp and Mimpen, 1979). Routine clinical tests include SRT primarily for the purpose of validating pure-tone threshold measurements (“cross-check principle”). The relationships between pure-tone average (PTA) threshold at 0.5, 1, and 2 kHz (PTA512) were assessed at baseline as a cross-check (based on the significant correlation described by Dobie and Sakai, 2001); SRT scores were not further analyzed.

#### Word recognition score (WRS)

Word Recognition Score (WRS) testing is supra-threshold testing during which participants attempt to correctly identify monosyllabic words, which are more difficult to identify than the spondee words used in SRT testing. Clinically, WRS is used to evaluate an individual's maximum speech understanding in an ideal listening environment (Dirks et al., 1977; Gelfand, 2001; McArdle and Hnath-Chisolm, 2014). Because understanding sound is more difficult than detecting sound, supra-threshold speech-based tests have been suggested to have the potential to distinguish audibility from intelligibility (Soli, 2008; Brungart et al., 2014). As part of the standard clinical battery used here, WRS was determined based on the number of correctly reported Northwestern University Auditory Test Number 6 (NU-6) words; recorded words were presented in quiet via the GSI Audiostar Pro. The NU-6 word list was presented at 40-dB above the participant's SRT; 25 words were presented to each ear. Although WRS is typically obtained at an intensity level intended to achieve the individual's maximum recognition ability (commonly abbreviated PBmax), this creates a problem with the use of these tests in research studies that include normal hearing participants as there is a ceiling effect in which normal-hearing listeners do uniformly well given the 40 dB SNR (see review by Le Prell and Clavier, 2017). The intensity level for the test is frequently set at a predetermined sensation level relative to the SRT or PTA threshold (Gelfand, 2001), with 40 dB SL being common (Martin et al., 1994). Based on the robust performance across participants and test sessions, there was no effort to systematically analyze the WRS data collected from the participants.

#### Words-in-noise (WIN) test

Speech-in-noise scores were assessed using the Words-in-Noise (WIN) test on the GSI Audiostar Pro following the procedures established by Wilson et al. (2003; for review, see Wilson, 2011). This test uses a subset of the NU-6 words spoken by a female speaker, with words presented in multi-talker babble composed of 6 female voices. The babble is fixed at 80-dB SPL as per Wilson et al. (2003), Wilson (2011). Target word level begins at 104-dB SPL and decreases in 4-dB steps from 104- to 80-dB SPL, providing 5 words at signal-to-babble (S/B) ratios that decrease from 24 (easiest) to 0 (most difficult). The primary performance metric is the 50% correct point, or dB S/B threshold, calculated using the equation dB S/B = 26 − (0.8 × N), with N defined as the total number of correct words across all conditions (for review, see Wilson, 2011). There are two 35-word lists with established equivalent recognition performance (Wilson and McArdle, 2007; Wilson et al., 2007a). There are 3 different randomization options for each of these lists; the randomization options were varied across the 3 test sessions in order to avoid practice effects. Wilson and McArdle (2007) defined 3.5 dB-S/B as a clinically meaningful difference between scores (corresponding to a difference of approximately 4 words out of the 35 words presented).

#### Electrophysiology

Two-channel ECochG data were collected using an Interacoustics Eclipse EP25 following the procedures described by Atcherson and Stoody (2012). The most common two-channel setup uses simultaneous ipsilateral and contralateral recording sites, with each ear serving as the inverting input for separate differential amplifiers. However, because the contralateral ear recordings were not analyzed in this study, the data generated via the two-channel setup are essentially equivalent to one-channel data collection; a two-channel setup was used to avoid the introduction of error in switching the electrode montage from right ear recordings to left ear recordings. Waveform repeatability was established during each test session at 70-, 80-, and 90-dB nHL for click, and 2, 3, and 4 kHz tone burst stimuli [Blackman, 5 cycles (termed “sines” within Eclipse stimulus parameters)]. Parameters were configured for alternating polarity, 11.7/s stimulus rate, and 500 sweeps of averaging. Etymotic ER3-26A gold electrodes (tiptrodes) were placed inside the ear canals, and Multipurpose Cloth electrodes (Oaktree Products, Inc.) were positioned in the standard adult diagnostic clinical configuration with non-inverting and ground electrodes stacked with spacing at midline high forehead (Fz). Electrode surface area was prepared with NuPrep and electrodes were prepared with Sanibel Lectron II conductivity gel. Action potential (AP) amplitude and summating potential (SP) amplitude were independently scored for each waveform by two different reviewers, with amplitude automatically calculated by the Interacoustics Eclipse EP25 system after peak marking. Although AP amplitude was easily identified across waveforms with scoring highly consistent across reviewers, SP amplitude was not as readily identified, and scoring was more variable. Variability in SP scoring across reviewers is well documented (see Roland and Roth, 1997). Discrepancies were resolved subsequent to review by a licensed audiologist after limiting the dataset to the 90 dB nHL waveforms, in which SP was clearest. SP was identifiable in all stimulus conditions (click, and 2, 3, and 4 kHz tonebursts) in 44% of left ears and 45% of right ears. The reviewers were masked with respect to L_Aeq8760_ and acute recreational noise dose while analyzing and marking waveforms, but the session at which the waveform was collected (baseline, next day, next week) was not masked.

### Statistical analyses

An initial series of analyses included comparisons of data from the right and left ears. These comparisons typically used two-way ANOVA with ear and frequency as dependent variables, although in the case of the WIN test, the signal to babble ratio (ranging from 0 to 24) was assessed in place of frequency. Statistical tools within SigmaPlot version 13.0 were used. SigmaPlot automatically handles the missing data by using a general linear model approach. This approach constructs hypothesis tests using the marginal sums of squares (also commonly called the Type III or adjusted sums of squares). SigmaPlot tests normality of the data distribution using the Shapiro-Wilk Normality Statistic with a criterion of *p* = 0.05. Equal variance assumptions are also tested using *p* = 0.05. There were cases in which one or both of these criteria were violated during the ANOVA tests. In those cases, one-way ANOVA on ranks was used instead, with analyses completed within frequencies. Because the DPOAE and ABR data were repeated within sessions, the data from the first and second runs were first compared using paired *t*-tests or Wilcoxon sign tests as appropriate (based on the outcome of the normality tests), and then the average of the two runs was used within the comparisons of the right and left ears. Although there were robust, statistically significant differences across frequencies and across dB S/B conditions, the ears were not systematically different; therefore, the average data values from the right and left ears were used in all subsequent analyses. Use of the average value despite some small right vs. left ear differences was explicitly intended to prevent inappropriate inflation of study power. Genetics, diet, smoking, cardiovascular disease, and most types of recreational noise exposure would be expected to affect both ears relatively equally, and thus the right and left ears are not independent. Although noise exposure might be asymmetric, particularly in the case of firearms, firearm use was rare (*n* = 3 female participants), and there was no evidence of asymmetric function in this small number of participants with a history of firearm use.

The second set of analyses assessed potential differences between males and females; these analyses used the averaged data from the right and left ears. These comparisons typically used two-way ANOVA with sex and frequency as dependent variables, although in the case of the WIN test, the signal to babble ratio (ranging from 0 to 24) was assessed in place of frequency. In those cases in which data were not normally distributed, one-way ANOVA on ranks was used instead, with analyses completed within each frequency. The Shapiro-Wilk test was used to assess normality of the distribution and the Brown-Forsyth Test was used to assess compliance with equal variance requirements. If either test was failed, then non-parametric tests were used. For comparisons of noise exposure, comparisons were via *t*-test if the normality and variance requirements were met, and were via Mann-Whitney Rank Sum tests if these conditions were not met.

To assess relationships between retrospective noise history (L_Aeq8760_) and auditory function at baseline, a series of correlation analyses were completed. Pearson correlation was used when data were normally distributed, and Spearman correlation was used in those cases where data were not normally distributed, as noted below. The Pearson correlation coefficient (R) is reported for parametric analysis, and the Spearman Rho (ρ) correlation coefficient is reported for non-parametric analysis. Linear regression lines of best fit are shown for data sets that were amenable to parametric analysis, and non-linear regression lines of best fit are shown for data sets that required non-parametric analysis.

Finally, multiple regression was used to assess the potential relationships between previous noise exposure (estimated using L_Aeq8760_) and auditory metrics to determine if functional outcomes could be predicted by noise history and other important variables (e.g., age, sex, and related functional test data). The analysis of the relationship between SP/AP ratio and L_Aeq8760_ was limited to the subset of waveforms in which both SP and AP could be readily identified. Multiple regression was completed within IBM SPSS Statistics version 23.

Statistical significance was defined as *P* < 0.05 for all analyses; when multiple pair-wise comparisons were required, statistical correction for multiple pair-wise comparisons was completed using Bonferroni correction. The Bonferroni correction compensates for the increase in risk of Type I errors associated with multiple pair-wise comparisons by testing each individual pair at a significance level of alpha/mu, where alpha is the desired overall alpha level (here, 0.05) and mu is the number of pair-wise comparisons completed. The Bonferroni correction can be too conservative if there are a large number of comparisons to be made.

## Results

### Comparisons of males vs. females

#### Previous 12-months noise exposure (L_Aeq8760_): no differences between males and females

Across participants, the average L_Aeq8760_ score obtained from the retrospective noise survey was 79.6 (SD = 4.3), with values ranging from 63.9 to 87.1. The mean L_Aeq8760_ score for males was 80.2 (SD = 2.9, range = 74.3–85.0). The mean L_Aeq8760_ score for females was 79.2 (SD = 5.0, range = 63.9–87.1); the female with the L_Aeq8760_ score of 63.9 was an outlier, as all other participants had L_Aeq8760_ scores of 72.4 or greater. Males were compared to females using a Mann-Whitney Rank Sum Test; there was no statistically significant difference with respect to retrospective noise exposure history assessed using L_Aeq8760_.

With the exception of the one female who reported very little noise exposure, the distribution of L_Aeq8760_ scores was highly similar to that reported by others. The range of L_Aeq8760_ noise scores was 64–84 for females and 64–88 for males in Megerson (2010), 67–83 for females and 70–82 for males in Stamper and Johnson (2015a), and 64–84 for females and 68–87 for males in Fulbright et al. (in press). Recent data from Spankovich et al. (2017) are also similar, with a range of scores from 66 to 83 for both male and female participants in this cohort. Taken together, the range of noise exposures experienced by this participant cohort is similar to (generally overlaps with) the range of noise exposures reported for young adult populations recruited on different campuses by different research teams. Although there was no effort to perform a statistical comparison of noise exposures across studies, the similar distributions of the exposure data across studies suggest the current cohort is not systematically different from other samples recruited by others. Individual L_Aeq8760_ scores were used as the basis for all analyses assessing potential effects of noise exposure history on different auditory metrics.

#### Pure-tone threshold sensitivity: males poorer than females at baseline

**Figure 1:**
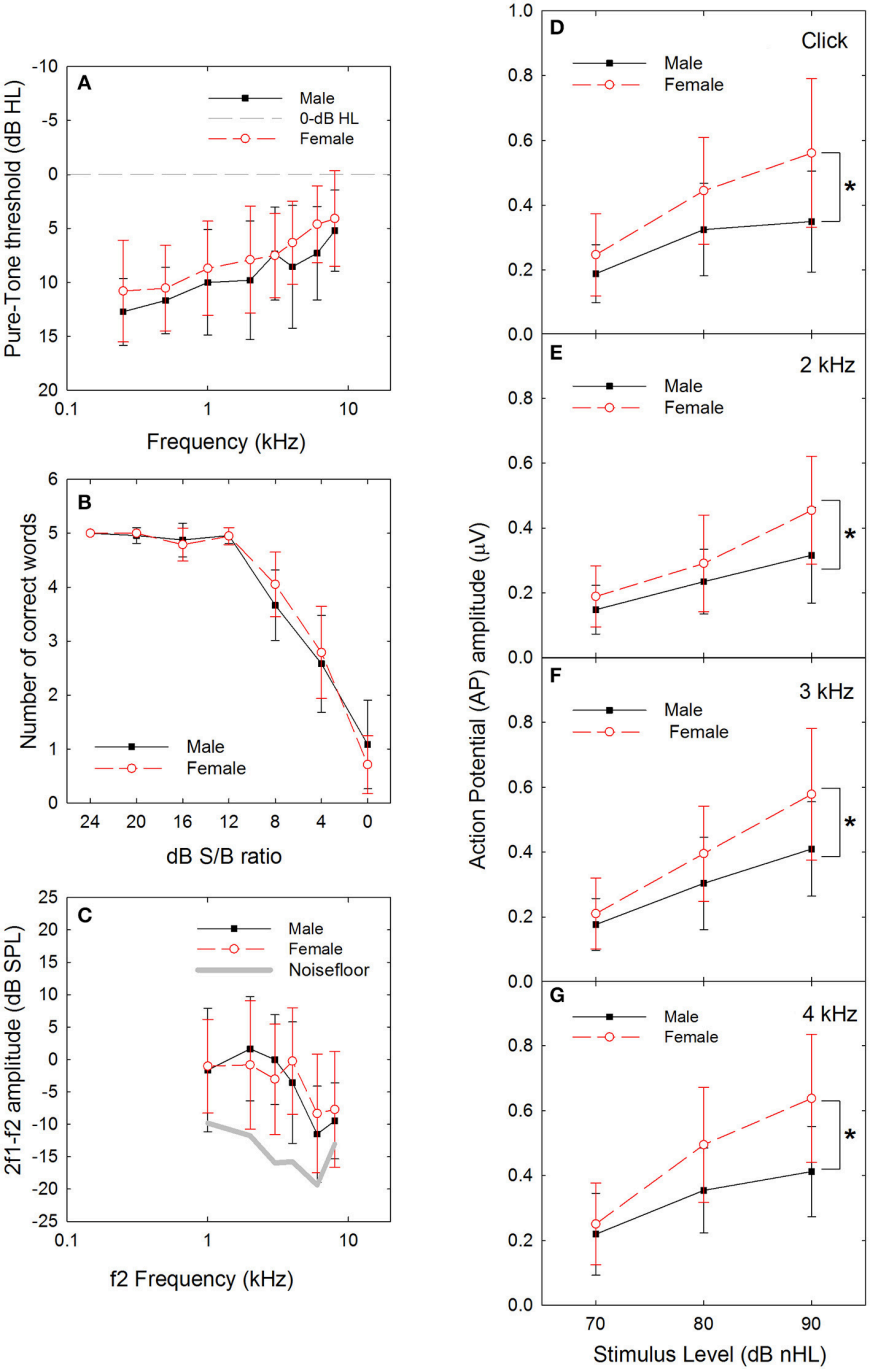
**(A)** There was a statistically significant difference in threshold at baseline, as a function of sex (male vs. female), with males having slightly poorer thresholds. Dashed line indicates 0-dB HL reference. **(B)** There were no statistically significant differences in performance within any of the signal-to-babble (dB S/B) conditions as a function of sex (male vs. female). **(C)** There was no statistically significant difference in distortion product otoacoustic emission (DPOAE) amplitude as a function of sex (male vs. female). **(D)** There were statistically significant differences in sound-evoked action potential (AP) amplitude as a function of sex (male vs. female), with females having significantly larger amplitudes compared to males with *p*-values less than 0.01 at 90 dB nHL levels (see asterisks) for clicks (**D**, *P* = 0.002) and tonebursts at 2 kHz (**E**, *P* = 0.006), 3 kHz (**F**, *P* = 0.004), and 4 kHz (**G**, *P* < 0.001). Sex differences at 80 dB nHL were statistically significant with *p*-values less than 0.05 for click and toneburst stimuli at 3 and 4 kHz, but not 2 kHz (click: *P* = 0.021; 2 kHz: 0.224; 3 kHz: *P* = 0.045; 4 kHz: *P* = 0.007). Data are mean ±1 SD.

The potential for threshold differences associated with sex was evaluated using two-way ANOVA with sex and frequency as independent variables. Both the normality and equal variance requirements were satisfied. There were statistically significant main effects for sex (*F* = 7.292, *df* = 1,247, *P* = 0.007) and frequency (*F* = 9.390, *df* = 1,7, *P* < 0.001), with no statistically significant interaction. Male thresholds were approximately 1–3 dB poorer than female thresholds across frequencies (see Figure 1A). Although there was adequate power to detect the main effect for sex, none of the Bonferroni-corrected pairwise comparisons were statistically significant when male and female thresholds were compared within frequencies. The overall small but statistically significant main effect for sex observed here replicates small but statistically significant differences in other cohorts in which males have had slightly poorer thresholds than females (Niskar et al., 1998; Serra et al., 2005; Kim et al., 2009; Shah et al., 2009; Shargorodsky et al., 2010; Le Prell et al., 2012, 2016; Spankovich et al., 2014). However, it is possible that the sex differences reported here and by others are an artifact of the study size as other studies have found no statistically significant differences as a function of sex (Henderson et al., 2011; Sekhar et al., 2011).

#### Words-in-noise (WIN) test: no differences between males and females at baseline

The potential for differences in performance on the WIN test as a function of sex was evaluated using two-way ANOVAs for the total number of words correct and the number of words correct within dB S/B conditions. There was a statistically significant effect of SNR (*F* = 299.151, *df* = 6,216, *P* < 0.001), with poorer performance as SNR decreased, but no statistically significant effect of sex (*F* = 0.117, *df* = 1,6, *P* = 0.733) was observed, nor were there any statistically significant interactions. Because the normality and equal variance tests were failed, a series of one-way ANOVAs (for data that met normality requirements) and ANOVA on Rank tests (for data that failed to meet normality requirements) were performed to assess potential sex differences within dB S/B conditions. There was no statistically significant effect of sex within 0 dB or 4 dB S/B conditions based on one-way ANOVA, and no statistically significant effect of sex within 8–24 dB S/B conditions based on one-way ANOVA on Rank tests. Participant performance was normally distributed at the most difficult listening conditions, but was skewed toward 100% correct within the easier signal-to-babble conditions (see Figure 1B). Similarly, there were no statistically significant sex differences on overall performance measures, including total number of words correct and dB S/B threshold, when one-way ANOVAs were completed (not shown).

#### Distortion product otoacoustic emission (DPOAE) amplitude: no differences between males and females at baseline

After averaging the data across run 1 and run 2, and for the left and right ears, a series of two-way ANOVAs with sex and frequency as independent variables were completed. There was a statistically significant main effect for frequency (*F* = 10.571, *df* = 5,185, *P* < 0.001) but not for sex (*F* = 0.261, *df* = 5,185, *P* = 0.610; see Figure 1C), and there was no statistically significant interaction. In general, Bonferroni-corrected pair-wise comparisons revealed that DPOAE response amplitude was larger at 1, 2, 3, and 4 kHz than at 6 and 8 kHz responses. Both the normality and the equal variance test requirements were met.

#### Action potential amplitude: statistically significant differences between males and females at baseline

Female AP amplitude was consistently larger than male AP amplitude at higher sound levels (see Figures 1D–G). To identify the statistical reliability of the differences between males and females, a three-way ANOVA with signal (click, 2, 3, or 4 kHz), level (70, 80, or 90 dB nHL), and sex (male vs. female) was performed. There were statistically significant main effects for signal (*F* = 15.480, *df* = 3,368, *p* < 0.001), level (*F* = 137.659, *df* = 2,368, *p* < 0.001) and sex (*F* = 71.936, *df* = 1,368, *p* < 0.001). In addition, there was a statistically significant interaction between sound level and sex, with males and females being statistically significantly different within the 80 (*t* = 4.593, *p* < 0.001) and 90 (*t* = 8.322, *p* < 0.001) dB nHL levels, but not at the 70 dB nHL level (*t* = 1.776, *p* = 0.077). Because the normality and equal variance tests were failed, a series of one-way ANOVA on ranks were used within signal x level conditions to confirm the statistical significance of the differences as a function of sex. As seen in Table 1, there were no statistically significant sex-related differences in ABR amplitude at 70 dB nHL. Statistically significant differences emerged at 80 dB nHL for several stimulus conditions (click: *P* = 0.021, 3 kHz: *P* = 0.045; 4 kHz: *P* = 0.007). Differences between males and females were statistically significant for all stimuli at 90 dB nHL (click: *P* = 0.002, 2 kHz: *P* = 0.006; 3 kHz: *P* = 0.004; 4 kHz: *P* < 0.001). If the statistical criterion is arbitrarily increased from 0.05 to 0.01 given the increased risk of Type I errors within the series of one-way ANOVAs (which are not corrected for pair-wise comparisons), then the statistically significant sex-related differences are generally limited to 90-dB nHL.

**Table 1:** ANOVA results for AP amplitude analyses, comparing males versus females.

	**70 dB nHL**	**80 dB nHL**	**90 dB nHL**
Click	*F* = 3.541, *df* = 1.30, *P* = 0.07	*F* = 5.977, *df* = 1.30, *P* = 0.021^*^	*F* = 11.342, *df* = 1.30, *P* = 0.002^*^
2 kHz	*F* = 2.749, *df* = 1.30, *P* = 0.108	*H* = 1.480 with 1 degrees of freedom (*P* = 0.224)	*F* = 8.699, *df* = 1.30, *P* = 0.006^*^
3 kHz	*H* = 1.032 with 1 degrees of freedom. (*P* = 0.310)	*F* = 4.382, *df* = 1.29, *P* = 0.045^*^	*F* = 9.634, *df* = 1.29, *P* = 0.004^*^
4 kHz	*F* = 0.633, *df* = 1.30, *P* = 0.433	*F* = 8.457, *df* = 1.30, *P* = 0.007^*^	*H* = 12.167 with 1 degrees of freedom (*P* = < 0.001^*^)

*The only statistically significant main effect was a main effect of sex, with females having larger AP amplitudes than males at higher presentation levels. Data are one-way ANOVA for those data sets in which normal distribution and equal variance requirements were met and one-way ANOVA on Ranks if parametric test requirements were not met*.

* *P < 0.05*.

### Relationships between previous 12-months noise exposure (L_Aeq8760_) and function

Multiple linear regression was used to assess whether retrospective noise history (based on the self-reported data used to calculate L_Aeq8760_) reliably predicts functional (audiologic) outcomes at baseline, including threshold, DPOAE amplitude, AP amplitude, SP/AP ratio, and WIN threshold. Each regression model included the specific functional outcome measured at the baseline visit as the dependent variable (DV), with independent variables (IVs) in each model specifically including retrospective self-reported noise history (L_Aeq8760_), age, sex, and related functional tests (i.e., DPOAE amplitude, audiometric threshold) measured at the baseline visit. Ear was not included as a predictor, as the initial analyses did not reveal statistically significant ear-related differences. The results of all models are provided in Table 2, with statistically significant models indicated with an asterisk. All statistical analyses were per the following strategy.

**Table 2:** Multiple regression models evaluated are listed below.

**Dependent Variable**	**Independent Variables**	***F***	***df***	***P***
DPOAE 1 kHz	Age, Sex, L_Aeq8760_	0.75	3, 22	0.53
DPOAE 2 kHz	Age, Sex, L_Aeq8760_	1.03	3, 22	0.40
DPOAE 3 kHz	Age, Sex, L_Aeq8760_	1.17	3, 22	0.34
DPOAE 4 kHz	Age, Sex, L_Aeq8760_	1.37	3, 22	0.28
DPOAE 6 kHz	Age, Sex, L_Aeq8760_	0.23	3, 22	0.88
DPOAE 8 kHz	Age, Sex, L_Aeq8760_	1.27	3, 22	0.32
1 kHz threshold	Age, Sex, L_Aeq8760_,DPOAE 1 kHz	4.09	4, 21	0.01^*^
2 kHz threshold	Age, Sex, L_Aeq8760_,DPOAE 2 kHz	1.89	4, 21	0.15
3 kHz threshold	Age, Sex, L_Aeq8760_,DPOAE 3 kHz	1.05	4, 21	0.41
4 kHz threshold	Age, Sex, L_Aeq8760_,DPOAE 4 kHz	3.47	4, 21	0.03^*^
6 kHz threshold	Age, Sex, L_Aeq8760_,DPOAE 6 kHz	1.76	4, 21	0.18
8 kHz threshold	Age, Sex, L_Aeq8760_,DPOAE 8 kHz	0.58	4, 21	0.68
AP – 2 kHz stimulus	Age, Sex, L_Aeq8760_,DPOAE 2 kHz, 2 kHz threshold	0.34	5, 20	0.88
AP – 3 kHz stimulus	Age, Sex, L_Aeq8760_,DPOAE 3 kHz, 3 kHz threshold	0.59	5, 19	0.71
AP – 4 kHz stimulus	Age, Sex, L_Aeq8760_,DPOAE 4 kHz, 4 kHz threshold	1.55	5, 20	0.22
AP – click stimulus	Age, Sex, L_Aeq8760_,average DPOAE 2-4 kHz, PTA234	1.44	5, 20	0.25
SP/AP – 2 kHz stimulus	Age, Sex, L_Aeq8760_,DPOAE 2 kHz, 2 kHz threshold	2.03	5, 8	0.18
SP/AP – 3 kHz stimulus	Age, Sex, L_Aeq8760_,DPOAE 3 kHz, 3 kHz threshold	0.17	5, 17	0.97
SP/AP – 4 kHz stimulus	Age, Sex, L_Aeq8760_,DPOAE 4 kHz, 4 kHz threshold	0.42	5, 13	0.83
SP/AP – click stimulus	Age, Sex, L_Aeq8760_,average DPOAE 2-4 kHz, PTA234	0.87	5, 7	0.55
WIN – 8 dB SNR	Age, Sex, L_Aeq8760_,DPOAE 4 kHz, PTA1234, AP – click stimulus	0.47	6, 19	0.82
WIN – 4 dB SNR	Age, Sex, L_Aeq8760_,DPOAE 4 kHz, PTA1234, AP – click stimulus	0.37	6, 19	0.89
WIN – 0 dB SNR	Age, Sex, L_Aeq8760_,DPOAE 4 kHz, PTA1234, AP – click stimulus	1.89	6, 19	0.14
WIN – Total score	Age, Sex, L_Aeq8760_,DPOAE 4 kHz, PTA1234, AP – click stimulus	0.95	6, 19	0.48

*There were no statistically significant effects of Sex, Age, or L_Aeq8760_. The models that were statistically significant (P < 0.05) are marked with an asterisk and the full results are provided in subsequent tables*.

* *P < 0.05*.

First, regression analysis was used to test if retrospective noise history predicted DPOAE amplitude. Each model included DPOAE amplitude (for each frequency 1–8 kHz) as the DV, and noise history (L_Aeq8760_), age, and sex as IVs. Results indicated the models to be non-significant for all frequencies (1–8 kHz), suggesting that none of the variables (noise history, sex, age) reliably predicted DPOAE amplitude. Next, regression was used to determine if retrospective noise history predicted audiometric threshold, using threshold (for each frequency from 1 to 8 kHz) as the DV, and noise history (L_Aeq8760_), age, sex, and DPOAE amplitude, with DPOAE frequency corresponding to the frequency of the threshold as IVs (e.g., 4 kHz threshold DV included when analyzing 4 kHz DPOAE IV). Results showed a statistically significant regression for the 1 kHz DV [*F*_(4, 21)_ = 4.09, *p* = 0.01 with *R*^2^ = 0.44] with DPOAE at 1 kHz as a significant predictor of the DV (see Table 3). The model predicting 4 kHz was also significant [*F*_(4, 21)_ = 3.47, *p* = 0.03 with *R*^2^ = 0.40] with DPOAE amplitude at 4 kHz as a significant predictor (see Table 4). All other models of audiometric threshold were found to be non-significant for all other frequencies. After correcting for multiple pair-wise comparisons using the Bonferroni procedure, the models in Tables 3, 4 did not meet the adjusted criteria for statistical significance.

**Table 3:** Multiple regression results for 1 kHz audiometric threshold at baseline.

	***B***	**SE**	***P***
Constant	−	23.29	0.31
Sex	0.16	2.36	0.56
Age	0.09	0.72	0.74
L_Aeq8760_	0.32	0.21	0.07
DPOAE at 1 kHz	−0.56	0.18	0.004^**^

*There were no statistically significant effects of Sex, Age, or L_Aeq8760_. The only statistically significant factor associated with 1 kHz audiometric threshold was DPOAE amplitude at 1 kHz*.

** *P < 0.01*.

**Table 4:** Multiple regression results for 4 kHz audiometric threshold at baseline.

	***B***	**SE**	***P***
Constant	−	27.17	0.20
Sex	0.07	2.77	0.80
Age	0.33	0.84	0.25
L_Aeq87860_	0.24	0.24	0.18
DPOAE at 4 kHz	−0.49	0.17	0.01^*^

*There were no statistically significant effects of Sex, Age, or L_Aeq8760_. The only statistically significant factor associated with 4 kHz audiometric threshold was DPOAE amplitude at 4 kHz*.

* *P < 0.05*.

**Figure 2:**
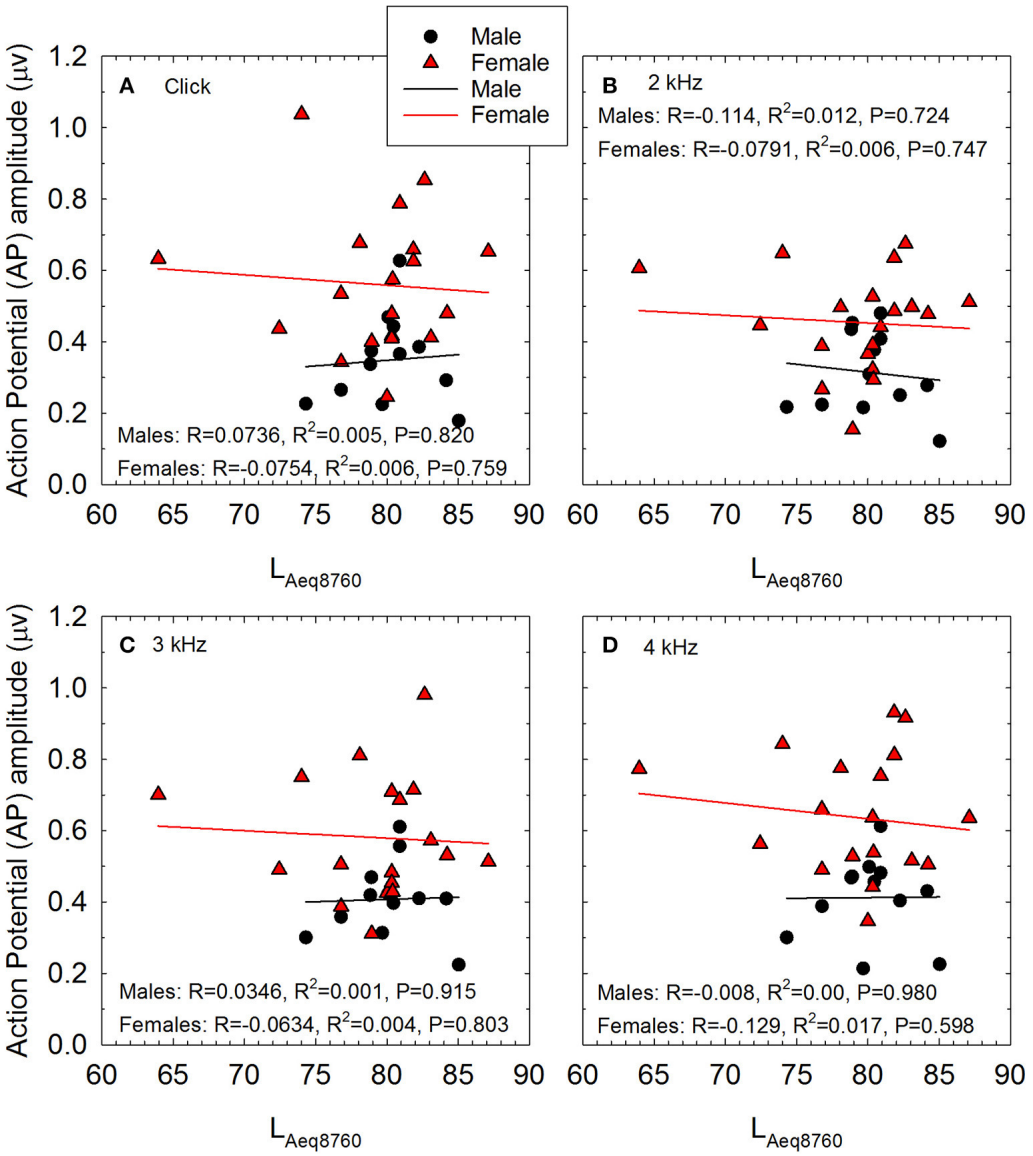
The relationship between self-reported noise exposure (calculated as L_Aeq8760_) and action potential (AP) amplitude is shown for male and female participants for stimuli including **(A)** clicks, **(B)** 2 kHz tone bursts, **(C)** 3 kHz tone bursts, and **(D)** 4 kHz tone bursts. All AP amplitude data were normally distributed. Pearson correlation analysis revealed no statistically significant relationships between self-reported noise history and AP amplitude within males or females. Lines of best fit are shown (Males: black symbols and regression lines; Females: red symbols and regression lines).

Regression was then utilized to test if noise history predicted AP amplitude. Each model included AP amplitude (for tone burst 2–4 kHz and click at 90 dB nHL input level) as the DV, and noise history (L_Aeq89760_), age, sex, DPOAE amplitude, and audiometric threshold as IVs. DPOAE amplitude and threshold frequency corresponded to frequency of AP input (e.g., 4 kHz AP DV included 4 kHz DPOAE and 4 kHz audiometric threshold IVs). For AP amplitude with click stimulus DV, DPOAE amplitudes and thresholds at 2–4 kHz were averaged and used as IVs, as the click stimulus has a broad frequency spectrum which stimulates the 2–4 kHz region of the cochlea as well as regions tuned to other frequencies (see Hall, 1992). Results indicated that the AP models were non-significant for all stimulus frequencies (see Figure 2 for correlation and line of best fit data). Additional regression analyses were performed to determine if noise history predicted SP/AP ratio. Each model included SP/AP ratio (for tone burst 2–4 kHz and click at 90 dB nHL input level) as the DV, and the same IVs as the previous analysis of AP. Results indicated that the models were not statistically significant at any stimulus frequencies (see Table 2). Because the analysis of the relationship between SP/AP ratio and L_Aeq8760_ was limited to the subset of waveforms in which both SP and AP could be readily identified, the sample size was smaller and power was reduced; as such, the lack of statistically significant relationships should be interpreted with caution. In cases in which a potential relationship between SP/AP ratio and function is observed, the interpretation of the SP/AP ratio requires careful consideration of the generators of both the SP and AP (see Discussion).

Finally, regression analysis was used to determine if noise history predicted WIN scores. Each model included WIN score (for each SNR from 0 to 8 dB S/B) as the DV, and noise history (L_Aeq8760_), age, sex, DPOAE amplitude (4 kHz input), audiometric threshold (PTA1234), and AP amplitude (click stimulus) as the IV. PTA1234 was selected based on Wilson et al. (2007b). Results indicated that the models were not statistically significant at any signal to noise ratio (see Table 2).

### Acute noise exposure at recreational events

**Figure 3:**
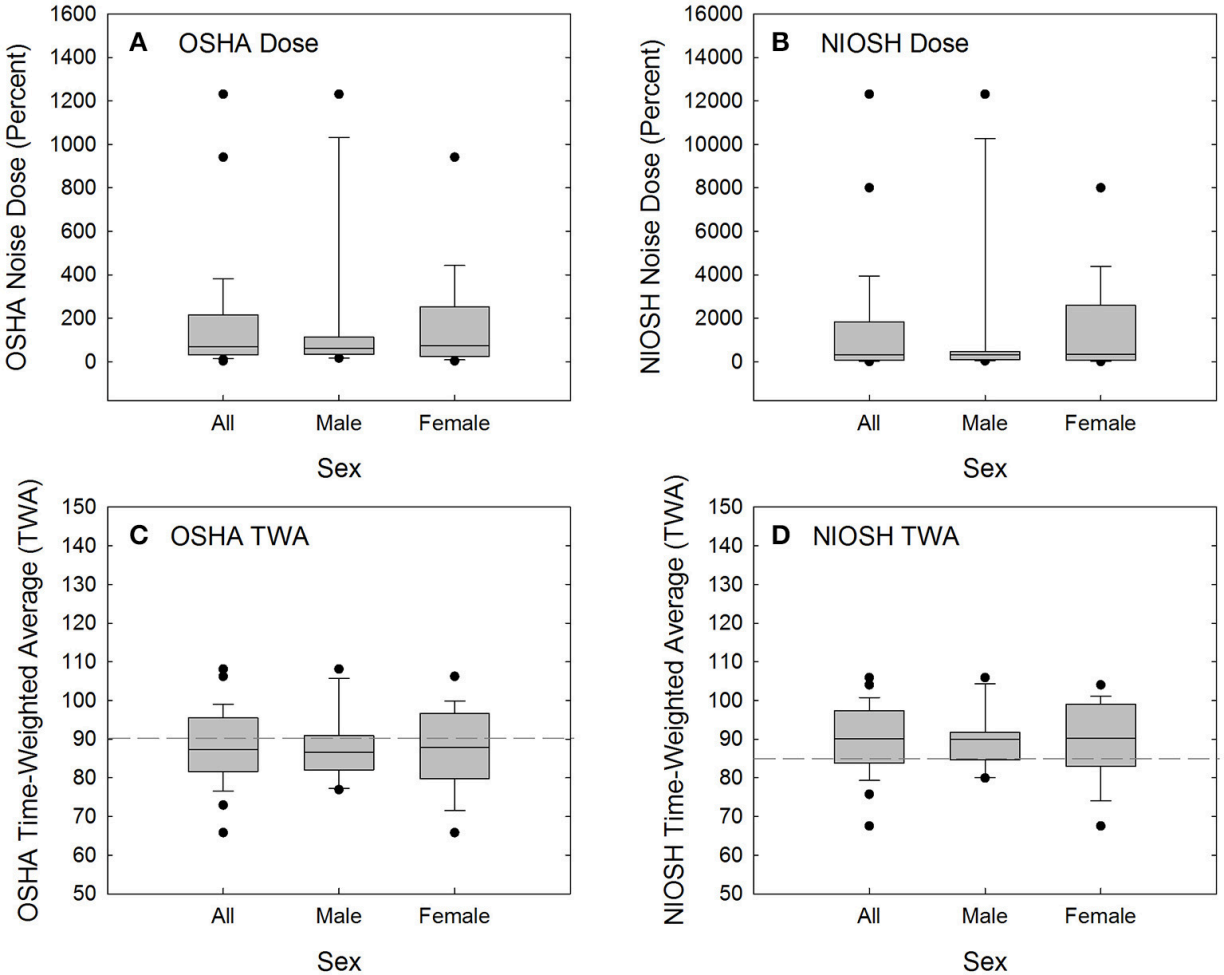
Acute noise exposure dose was calculated per 29 CFR 1910.95 (OSHA, 1983) **(A)** and per the recommended procedures suggested by NIOSH (1998) **(B)**. Calculated dose was converted to time-weighted average (8-h equivalent level) per 29 CFR 1910.95 **(C)** and the NIOSH recommended criteria **(D)**. By converting from dose to TWA, the effects of two outliers are reduced and the distribution is normalized. OSHA TWA is calculated based on 100% dose being equivalent to 8 h exposure to 90-dBA noise (dashed line in **C**). NIOSH TWA is calculated based on 100% dose being equivalent to 8 h exposure to 85-dBA noise (dashed line in **D**).

A total of 28 of the original 32 participants attended a recreational event that they deemed “loud,” and returned the day after the event for repeat audiometric testing (see Table 5 for event summary, sound level measurements, and duration of event attendance). Calculated using 29 CFR 1910.95, the average participant noise dose was 168.4 ± 276% (range 3.5–1,230.8%), based on event levels of 93.3 ± 7.8 dBA (range 73.1–104.2 dBA) and durations of 4.2 ± 3.5 h (range 1.5–16.0 h). There were two participants with 16-h attendance at a music festival with sound levels of 103–104 dBA; these two participants (one male, one female) had much higher doses than the other participants (see Figure 3A). Excluding these two outliers, the average recreational noise exposure was 92.7 ± 7.7 dBA (range 73.1–104.2 dBA) for 3.3 ± 0.9 h (range 1.5–4.5 h), yielding an event dose of 97.8 ± 92.5% (range 3.5–318.2%). There were 9 participants with doses of less than 50% (4 male, 5 female), 10 participants with doses of 50 to 100% (4 male, 6 female), and 9 participants with doses above 100% (3 male, 6 female). There was no statistically significant difference in OSHA exposure dose for males and females compared via Mann-Whitney Rank Sum Test (Mann-Whiney U statistic = 88.000, *P* = 0.814; Shapiro-Wilk Normality Test failed).

**Table 5:** Acute noise exposure.

**Participant ID**	**Sex**	**Event**	**Time (H)**	**Level**	**NIOSH Dose (%)**	**NIOSH TWA**	**OSHA Dose (%)**	**OSHA TWA**
004	F	Movie	2.25	73.1	1.8	67.5	3.5	65.8
012	M	Bar	3	84.2	31.2	79.9	16.3	76.9
028	M	Bar	3	91.9	185.2	87.7	49.2	84.9
032	M	Bar	2.5	94.9	308.6	89.9	62.5	86.6
008	F	Bar	3	104.2	3157.9	100.0	272.7	97.2
005	F	Bar/live music	3	96	476.2	91.8	85.7	88.9
006	M	Bar/live music	3	96	476.2	91.8	85.7	88.9
019	M	Bar/live music	6	93	476.2	91.8	113.2	90.9
022	F	Concert	3	80	11.8	75.7	9.4	72.9
007	F	Concert	3.5	83.3	29.7	79.7	16.6	77.0
016	M	Concert	3.75	83.6	33.9	80.3	20.4	78.5
014	F	Concert	1.5	89.7	55.6	82.4	18.8	77.9
018	F	Concert	3.75	86.8	71.0	83.5	31.0	81.5
009	M	Concert	3.5	88.2	91.6	84.6	33.0	82.0
003	F	Concert	5	89.4	173.0	87.4	62.5	86.6
020	M	Concert	2.5	93.5	204.9	88.1	54.3	85.6
002	F	Concert	2.5	93.5	223.2	88.5	54.3	85.6
017	M	Concert	3	95.7	441.2	91.4	85.7	88.9
001	F	Concert	4.5	97.5	1022.7	95.1	173.1	94.0
021	M	Concert	4	101.1	2105.3	98.2	235.3	96.2
029	F	Concert	3	102.5	2142.9	98.3	230.8	96.0
026	F	Concert	3	104	3030.3	99.8	272.7	97.2
027	F	Concert	3.5	103.9	3500.0	100.4	318.2	98.3
031	F	Dance event	4.5	91.8	271.1	89.3	73.8	87.8
013	F	Dance event	2.25	95.7	330.9	90.2	64.3	86.8
010	F	Dance event	3	96.5	535.7	92.3	100.0	90.0
024	F	3 day festival	16	101	8000.0	104.0	941.2	106.2
023	M	3 day festival	16	102.7	12307.7	105.9	1230.8	108.1

*Sound level measurements collected via app and duration of exposure as per participant report*.

Because NIOSH guidance (NIOSH, 1998) advocates more conservative exposure limits than OSHA regulations require (OSHA, 1983), the noise dose accrued at the event is higher when calculated based on NIOSH recommendations (see Table 5, Figure 3B). When noise dose was instead calculated using NIOSH recommendations of an 85-dBA relative exposure limit (REL) and using a 3-dB exchange rate, there were 5 participants with doses of less than 50% (2 male, 3 female), 3 participants with doses of 50 to 100% (1 male, 2 female), and 20 participants with doses above 100% (8 male, 12 female). There was no statistically significant difference in exposure assessed as NIOSH dose for males and females when compared via Mann-Whitney Rank Sum Test (Mann-Whiney U statistic = 88.000, *P* = 0.814; Shapiro-Wilk Normality Test failed).

Dose was converted to time-weighted average (the 8-h equivalent level) as shown in Figures 3C,D. OSHA TWA is calculated based on 100% dose being equivalent to 8-h exposure to 90-dBA noise, with a 5-dB exchange rate used for sound levels other than 90-dBA (see dashed line in Figure 3C). NIOSH TWA is calculated based on 100% dose being equivalent to 8-h exposure to 85-dBA noise, with a 3-dB exchange rate used for sound levels other than 85-dBA (see dashed line in Figure 3D). There was no statistically significant difference in OSHA TWA for males and females when compared via *t*-test (*t* = −0.0865 with 26 degrees of freedom, two-tailed *P*-value = 0.932; both Shapiro-Wilk Normality Test and Brown-Forsythe Equal Variance Test passed). Similarly, there was no statistically significant difference in NIOSH TWA for males and females when compared via *t*-test (*t* = −0.0590 with 26 degrees of freedom, two-tailed *P*-value = 0.953; both Shapiro-Wilk Normality Test and Brown-Forsythe Equal Variance Test passed).

A series of correlation analyses were used to assess potential linear relationships between acute exposure (OSHA TWA) and functional change. OSHA TWA was normally distributed. Pearson correlation was used when all data were normally distributed, and, Spearman correlation was used in those cases where a subset of the data were not normally distributed as noted below.

#### Acute noise-induced changes in pure-tone threshold sensitivity

After pre-noise baseline was established (Figure 1), most of the participants attended a loud event (*n* = 28). Thresholds were reassessed the day after the event (within 24 h of the event). The timing of the post-noise tests (i.e., the day after the loud event) was explicitly selected to parallel the timing in animal studies (Kujawa and Liberman, 2009; Lin et al., 2011; Wang and Ren, 2012; Hickox and Liberman, 2014; Fernandez et al., 2015; Jensen et al., 2015; Lobarinas et al., 2017). The final test 1-week later was used to assess recovery of any changes; 26 of the 28 participants returned for the final test.

**Figure 4:**
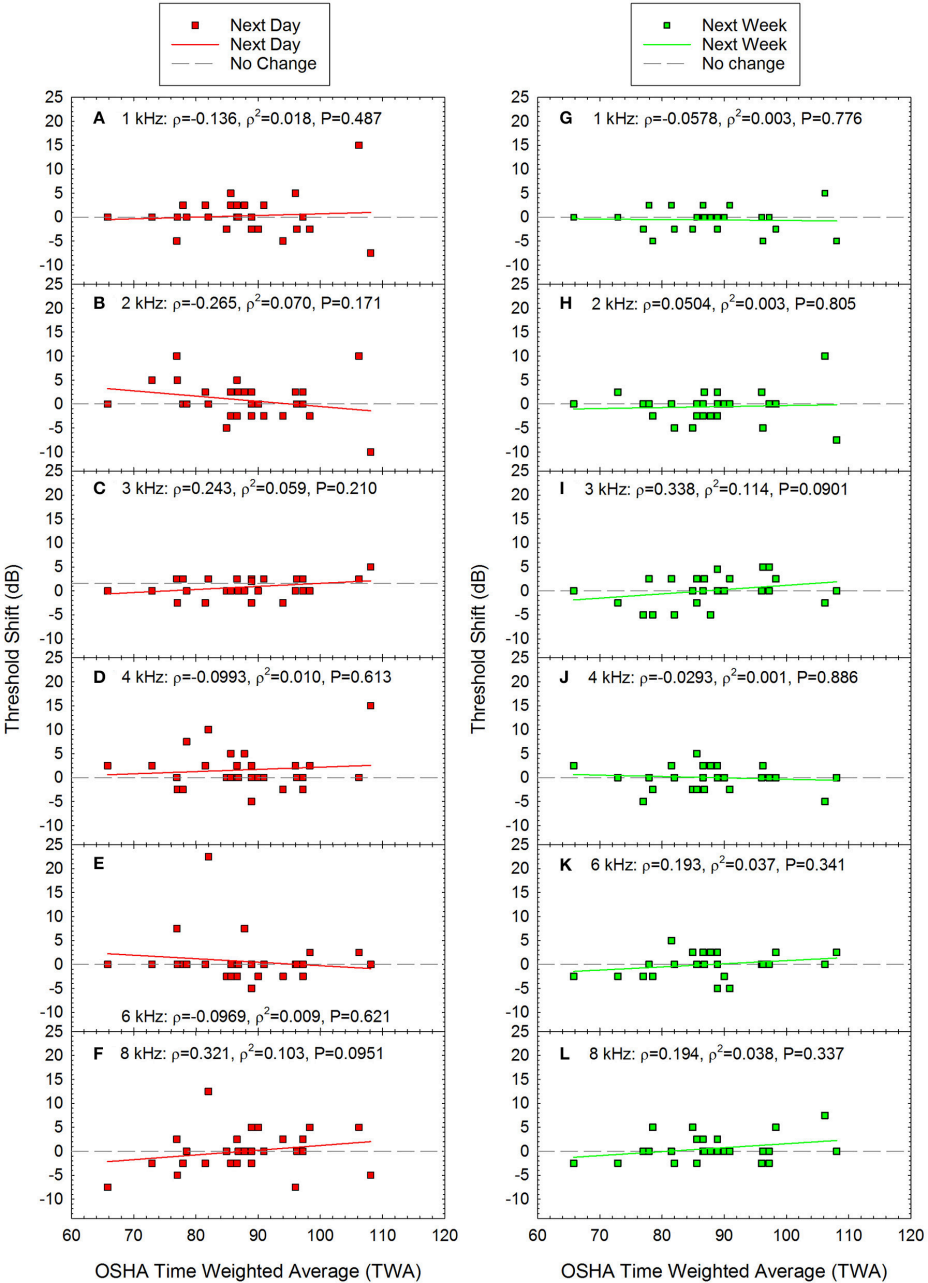
There were no statistically significant correlations between time-weighted-average (TWA) and threshold shift at any of the frequencies tested either the day after the loud event **(A–F)** or one week later **(G–L)**. Next day data are shown for **(A)** 1 kHz, **(B)** 2 kHz, **(C)** 3 kHz, **(D)** 4 kHz, **(E)** 6 kHz, and **(F)** 8 kHz. Next week data are shown for **(G)** 1 kHz, **(H)** 2 kHz, **(I)** 3 kHz, **(J)** 4 kHz, **(K)** 6 kHz, and **(L)** 8 kHz. Lines of best fit are shown.

TTS (calculated as the difference between the pre-noise threshold and the post-noise threshold) as a function of acute noise exposure is shown in Figures 4A–F. There was significant individual variability across participants, and the TTS data were not normally distributed. There was one participant with an average shift of 10 dB and three participants with threshold shifts greater than 10 dB; across these four participants, the frequency at which the shift was observed varied, including 1, 2, 4, and 6 kHz. At the 1-week test session, most participants had thresholds that were within ±5 dB of the original pre-noise baseline, although a small number of data points were more variable and were within ±10 dB relative to baseline (see Figures 4G–L). Spearman correlation was used to determine if there were any statistically significant relationships between exposure and threshold shift the day after the recreational activity. None of the correlations were statistically significant (see Figure 4 for scatterplots and Spearman Rho coefficient of determination).

#### Acute noise-induced changes in performance on the word-in-noise (WIN) test

**Figure 5:**
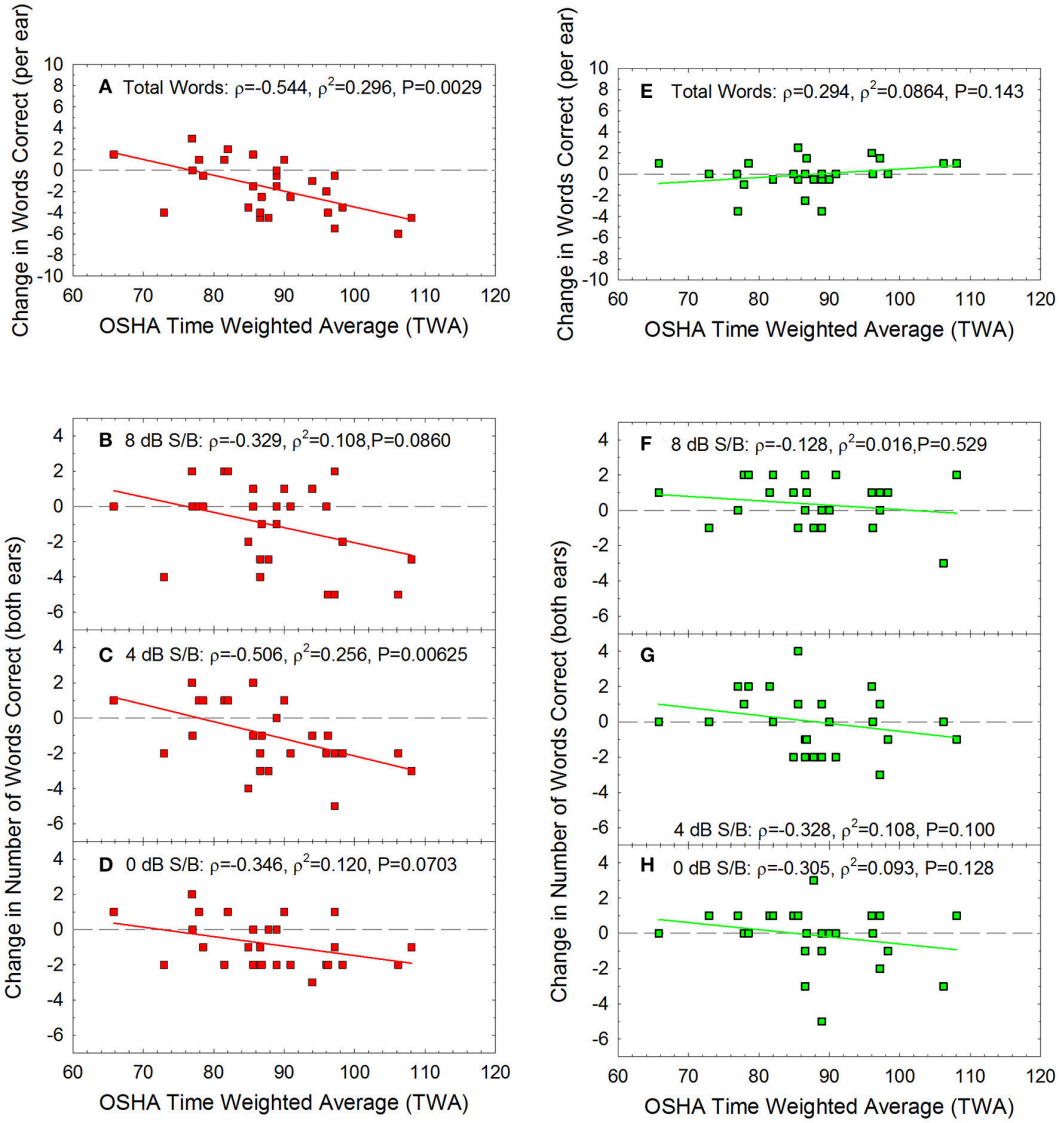
For the Words-in-Noise (WIN) test, the summed change in performance was calculated as the total number of additional words correct (positive scores) or incorrect (negative scores) at the post-tests, relative to baseline, the “next day” (red) and “next week” (green). There was a statistically significant correlation between noise exposure (TWA) and the number of words missed the day after the noise exposure **(A)**, with the largest changes being approximately 6 words per ear out of the 35-word test lists. There were no statistically significant decreases in performance at the 1-week test time **(E)**, with the greatest deficits being approximately 3 words out of the 35 word lists; this is not a clinically significant change in speech-in-noise performance. The biggest temporary changes in performance were observed at the most difficult listening conditions. There was a statistically significant correlation between noise dose and change in performance the day after exposure within the 4 dB S/B condition **(C)**, with the largest changes being approximately 6 words out of the 10 words total that were presented to the two ears. There were similar trends for temporarily poorer performance as a function of noise exposure at other signal to noise conditions including **(B)** 8 dB/SB and **(D)** 0 dB S/B, but these were not statistically significant relationships. No statistically significant changes were evident at the one-week post noise test within **(F)** 8 dB S/B, **(G)** 4 dB S/B, or **(H)** 0 dB S/B conditions. Lines of best fit are shown.

Change in performance on the WIN was calculated as the difference between pre-noise baseline performance (see Figure 1B) and post-noise performance. The average change in the summed performance across the 35-word lists is shown in Figures 5A,E, and the total change within each dB S/B conditions (5 words presented per ear per SNR condition, from 0 to 24 dB S/B) is shown for the more difficult SNR conditions, including 8 dB S/B (Figures 5B,F), 4 dB S/B (Figures 5C,D), and 0 dB S/B (Figures 5D,H) signal to babble ratios. There was significant individual variability, and the change in performance data were not normally distributed. Spearman correlation was therefore used to determine if there were any statistically significant relationships between acute noise exposure and change in WIN performance. The correlations were statistically significant for the overall change in performance the next day (maximum possible change in score = −35 words if performance went from 100% correct to 0% correct) and within the 4 dB S/B condition (maximum possible change in score = −10 words if performance for both ears went from 5 words correct to 0 words correct). At other SNRs, there were similar trends in which performance on the WIN the day after exposure appeared to decrease as a function of increasing recreational noise exposure, but the *P*-values for the other dB S/B conditions did not meet the criterion of *P* < 0.05. The predicted change in overall performance on the WIN 35-word list as a function of noise exposure at the next day test session shown in Figure 5A was: change in performance on WIN = 11.511 + (−0.150 × TWA). There were no statistically significant relationships between WIN score shifts and noise exposure at 1-week post-noise on the overall test or within dB S/B conditions. None of the individual participants met the clinically significant change criteria derived by Wilson and McArdle (2007) at the 1-week post-noise test time.

#### Acute noise-induced changes in distortion product otoacoustic emission (DPOAE) amplitude

**Figure 6:**
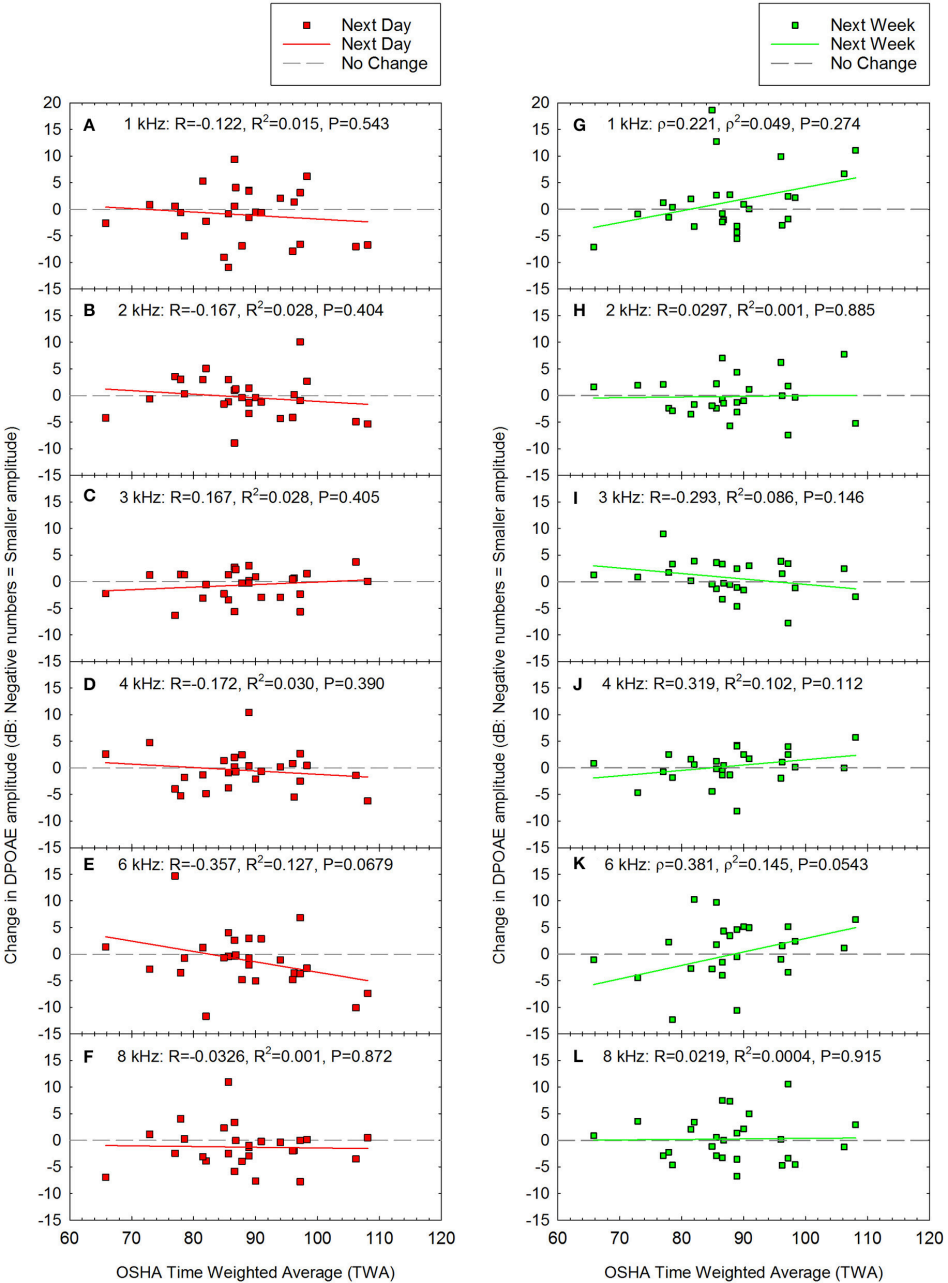
There were no statistically significant correlations between noise exposure (TWA) and changes in DPOAE amplitude either the day after the loud event **(A–F)** or one week later **(G–L)**. Next day data are shown for **(A)** 1 kHz, **(B)** 2 kHz, **(C)** 3 kHz, **(D)** 4 kHz, **(E)** 6 kHz, and **(F)** 8 kHz. Next week data are shown for **(G)** 1 kHz, **(H)** 2 kHz, **(I)** 3 kHz, **(J)** 4 kHz, **(K)** 6 kHz, and **(L)** 8 kHz. Although there was a trend for decreased amplitude at 6 kHz **(E)**, this was not statistically significant (*P* = 0.0679). Lines of best fit are shown.

Change in DPOAE amplitude was calculated as the difference between pre-noise DPOAE amplitude (see Figure 1C) and post-noise DPOAE amplitude at each test frequency. Change in DPOAE amplitude as a function of the acute noise dose is shown in Figure 6. The data were normally distributed at all frequencies for the next day data set, and for all but 1 and 6 kHz at the next week test. Pearson correlation was therefore used to determine if there were any statistically significant relationships between OSHA TWA and change in DPOAE amplitude except at 1 and 6 kHz at the next week test session, for which Spearman correlation was assessed. There were no statistically significant correlations.

#### Acute noise-induced changes in auditory brainstem response amplitude post-exposure

**Figure 7:**
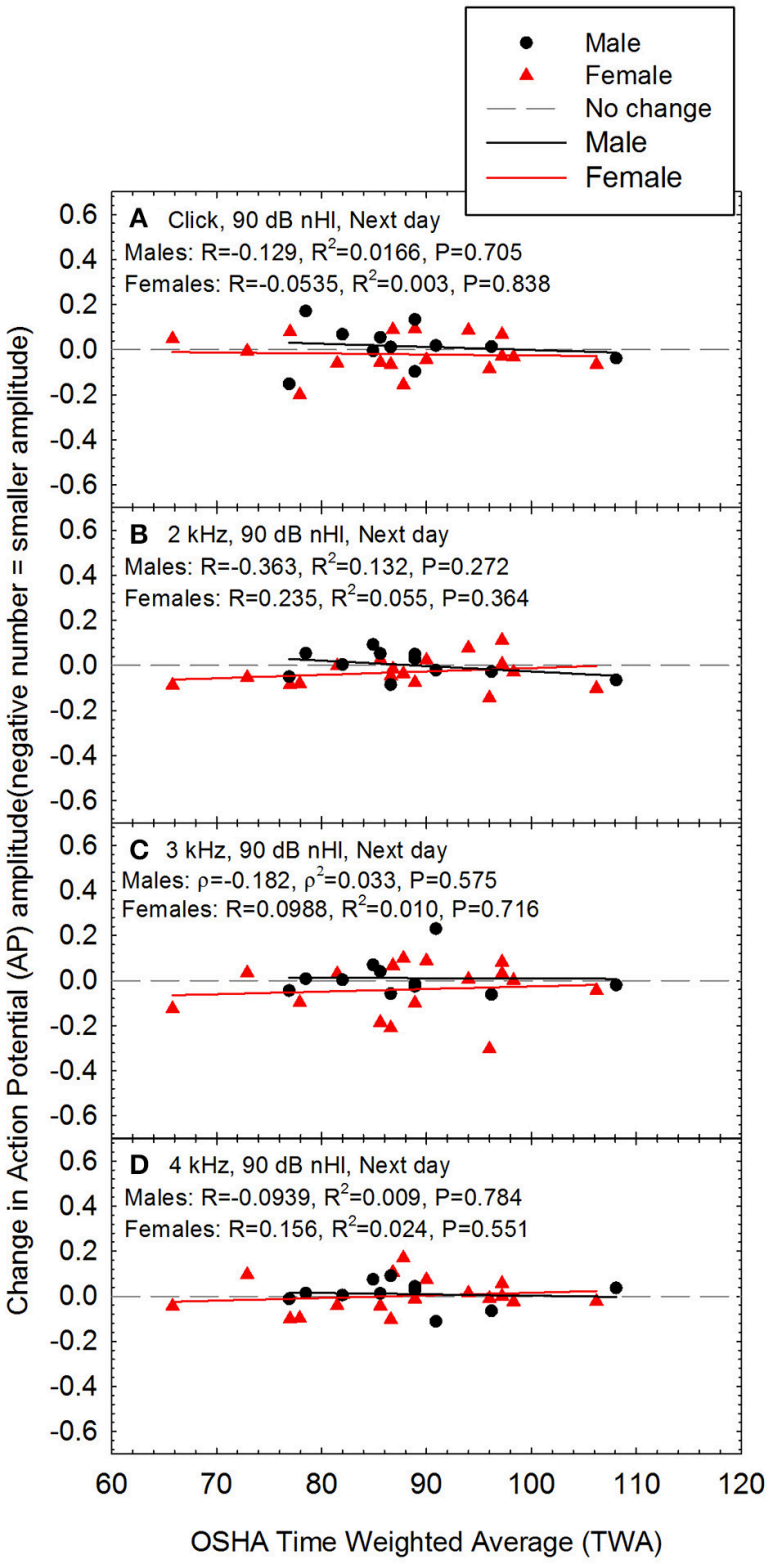
There was no evidence of a noise-induced decrease in sound-evoked AP amplitude regardless of whether the stimuli were **(A)** clicks, **(B)** 2 kHz tone bursts, **(C)** 3 kHz tone bursts, or **(D)** 4 kHz tone bursts; all data are for 90 dB nHL stimuli, as measured the day after the noise exposure. None of the relationships were statistically significant within males or females. Lines of best fit are shown.

Because there were statistically significant differences between males and females with respect to AP amplitude (see Figures 1E–G), changes in AP amplitude after noise exposure were analyzed separately for males and females. There was no statistically significant evidence of noise-induced decreases in AP amplitude, and there was no change at the individual level even in the two participants with the highest noise doses (see Figure 7). Noise exposure (TWA) and changes in AP amplitude data were both normally distributed within Female participants. Pearson correlation was used to assess whether there was any relationship between TWA and change in AP amplitude within Females. The TWA data was normally distributed within Male participants; the changes in AP amplitude were normally distributed at 2 and 4 kHz, and for clicks, but not for 3 kHz data. Therefore, Spearman correlation was used to assess whether there was any relationship between dose and change in AP amplitude within Males at 3 kHz, and Pearson correlation was used for the other analyses. There were no statistically significant relationships between noise exposure and changes in AP amplitude within males or females.

### Relationship between temporary threshold shift and other acute noise-induced changes

**Figure 8:**
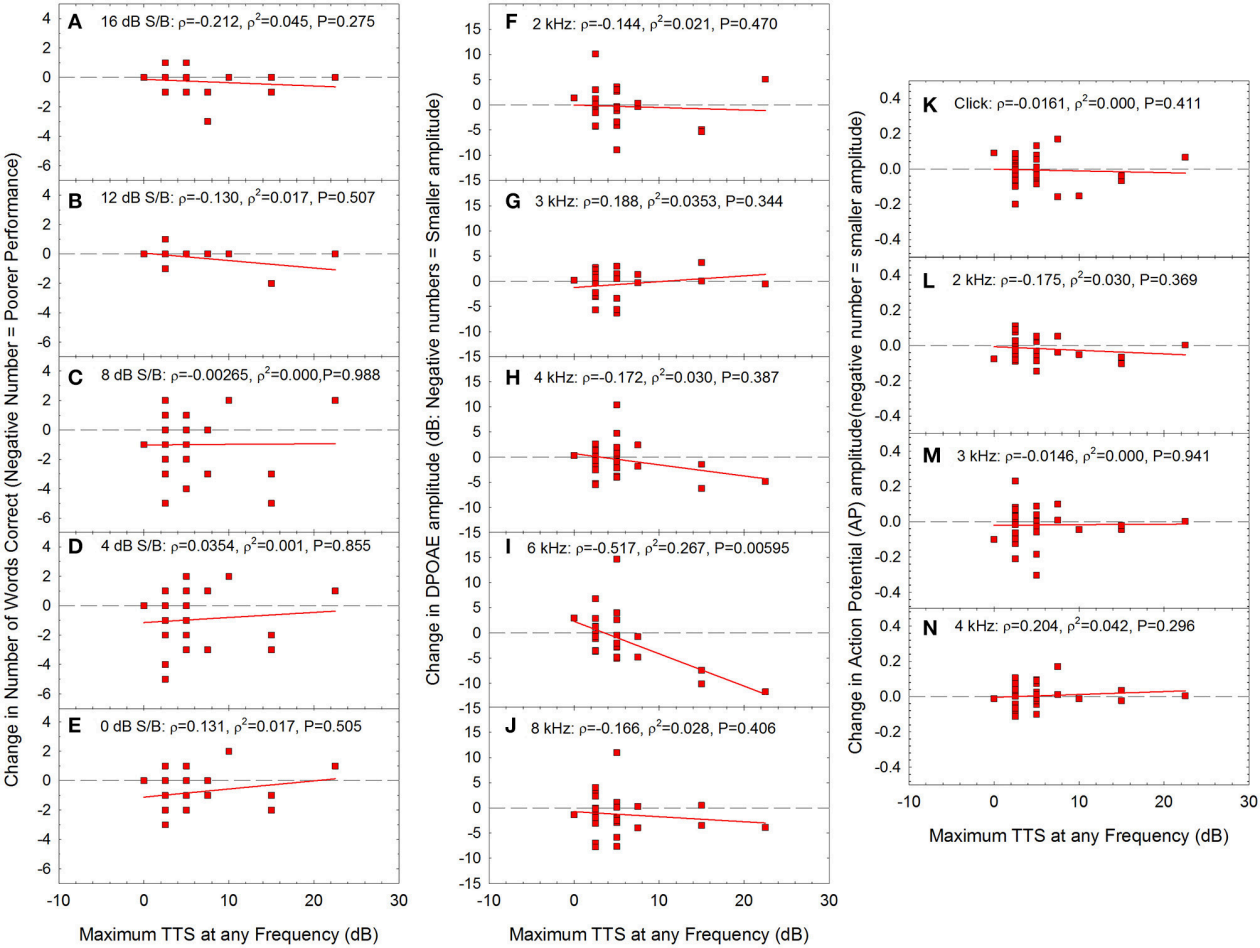
There were no statistically significant correlations between maximum TTS at any frequency and change in performance within any of the signal-to-babble conditions (**A**: 16 dB S/B; **B**: 12 dB S/B; **C**: 8 dB S/B; **D**: 4 dB S/B; **E**:0 dB S/B). There was a statistically significant correlation between maximum TTS at any frequency and change in DPOAE amplitude at 6 kHz **(I)** with no statistically significant relationships at other frequencies (**F**: 2 kHz; **G**: 3 kHz; **H**: 4 kHz; **J**: 8 kHz). There was no statistically significant relationship between maximum TTS at any frequency and change in AP amplitude. (**K**: click; **L**: 2 kHz; **M**: 3 kHz; **N**: 4 kHz). Lines of best fit are shown in all panels.

Across audiometric measures (see Figures 4–7), there was significant individual variability with respect to the effects of noise on auditory function. Some participants had seemingly more “tender” ears, with larger changes in function after relatively lower noise doses. Other participants had seemingly “tougher” ears, with smaller changes in function, despite relatively larger noise doses. Based on this, additional analyses were performed in which changes in performance on the WIN (Figures 8A–E), changes in DPOAE amplitude (Figures 8F–J), and changes in AP amplitude (Figures 8K–N) were assessed as a function of the maximum TTS measured at any frequency the day after the noise exposure. Because maximum TTS at any frequency was not normally distributed, Spearman Rank Order correlation was used to assess all potential relationships.

The only statistically significant relationship between TTS the day after the exposure and other metrics was DPOAE amplitude at 6 kHz (see Figure 8I). As TTS increased, there were increasing deficits in DPOAE amplitude at 6 kHz. Taken together, the data may suggest that in the participants that had the most severe TTS, the OHCs were the most vulnerable element, based on the data showing statistically significant decreases in OHC function at 6 kHz. Because these changes were limited to 6 kHz, and noise is expected to broadly affect the entire 3–6 kHz region, additional research will be necessary to more fully understand any underlying temporary damage to the cochlea. There were no statistically significant reductions in AP amplitude as a function of increasing TTS. Moreover, there were no reductions in AP amplitude within the small subset of individuals with TTS of 10 dB or more. Thus, while OHCs may have possibly been damaged in participants with the greatest TTS, there was no evidence of neural injury.

## Discussion

In the first part of this investigation, a retrospective analysis in which noise survey responses were used to compare previous noise exposure history to current auditory function, there was no evidence that a history of self-reported common recreational exposures resulted in audiometric, functional, or electrophysiological deficits. These data parallel Prendergast et al. (2017), Fulbright et al. (in press), and Spankovich et al. (2017), who evaluated three different normal-hearing young adult cohorts with varying amounts of recreational noise exposure history. In contrast to these four studies, ABR Wave-I amplitude was reported to be reduced in young adults (or, at least in young adult females) as a function of recreational noise exposure by Stamper and Johnson (2015a,b). Of note, none of these populations had significant occupational noise exposure histories or systematic exposure to loud music as rehearsing or performing musicians.

In contrast to the negative results from the above studies, Liberman et al. (2016) described statistically significant differences in extended high frequency (EHF) threshold sensitivity, word recognition performance in difficult listening conditions, SP amplitude, and the SP/AP ratio when high risk participants (15M, 7F; largely, college students enrolled in a music conservatory) were compared to low risk participants (4M, 8F; largely, college students enrolled in a communication sciences program). Bramhall et al. (2017) has also described deficits in ABR Wave-I amplitude as a function of noise exposure; they compared the amplitude of ABR Wave-I in civilians and military personnel without significant noise exposure to civilians who use firearms and military personnel with significant noise exposure (including firearm use). Taken together, the majority of data across retrospective studies now appear to be generally consistent in revealing no statistically significant relationships between common recreational noise exposure histories and ABR Wave-I (or AP) amplitude, whereas statistically significant relationships between firearm, blast, and other significant noise exposure and ABR Wave-I amplitude have emerged (Bramhall et al., 2017).

It is possible that statistically significant associations between AP (or, ABR Wave-I amplitude) and recreational noise history would emerge if larger cohorts were studied, which would increase power to detect subtle relationships. However, based on the observed Pearson R and Spearman Rho values of 0.15 or less across stimulus conditions (see Figure 2), new, prospective power analysis indicates that a sample size of 400 participants would be necessary to achieve 85% power to detect relationships of the size (i.e., *R* = 0.15) obtained in this retrospective analysis. Even if a large study with the power to detect small associations was conducted, it is not clear that the weak relationships indicated by *R* values of 0.15 would be clinically significant. As an alternative, study power presumably would be increased if additional higher-risk participants were included, assuming that the hypothesis that the strength of the observed relationships will increase as participants with increasing exposure are added is true. It is not yet clear if risk will increase relatively linearly along some graded continuum as noise exposure increases, or if there is some critical boundary at which risk of injury suddenly increases in an “all or nothing” fashion; a better understanding of this relationship is critically important with respect to the design of future studies and the eventual development of evidence-based risk criteria. Systematic manipulation of noise exposure using rodent models may provide some insight into these relationships and inform the design of human translational studies. Data from non-human primates are also likely to be necessary in order to understand risk across species, and would support additional inference related to human risk.

Most investigations assessing the potential for hidden hearing loss in humans have used NEQ-based approaches. These studies rely on an assumption that reports of noise exposure within the past 12-months provide information that is relevant and accurate. These studies further assume that exposure over the past 12-months is representative of previous lifetime noise history. If there was significantly more or less noise exposure within the past 12-months than in earlier years, the previous 12-month L_Aeq8760_ metric would provide limited utility for comparisons with current functional status. Fulbright (2016) used a variety of surveys to assess both L_Aeq8760_ and lifetime noise. No notable differences in outcomes were observed when current audiometric function was assessed as a function of L_Aeq8760_ or lifetime noise; however, in this young adult population, lifetime noise estimates tended to be reduced relative to previous year estimates. In other words, noise exposure as a young adult was increased relative to noise exposure in earlier childhood years. Thus, it cannot be assumed that every participant in every study has a 12-month noise history (and L_Aeq8760_) that is representative of their lifetime noise exposure history; careful interview is necessary to assure that there was no significant noise exposure in earlier years that would suggest a participant is at higher risk than their current L_Aeq8760_ might suggest.

We did not survey self-reported difficulties in noise. It is tempting to assume that self-reported difficulty listening in noise may be a useful measure, as this approach is now being used in large epidemiological studies that rely on survey data to assess hearing problems (see for example Curhan et al., 2012). The use of surveys may also resolve challenges related to the ceiling effects observed for some speech-in-quiet and speech-in-noise tests. Certainly, there is a lack of consensus regarding an accepted “gold standard” for speech-in-noise testing (for discussion, see Le Prell and Lobarinas, 2015; Le Prell and Brungart, 2016; Le Prell and Clavier, 2017). The data collected here used the WIN test, while Bramhall et al. (2015) collected data using the QuickSin, and Liberman et al. (2016) used the widely available NU6 words within a custom hearing-in-noise test which included the addition of time compression and reverberation to NU6 words to increase the difficulty of the standardized test. There is a need for standardized, quantitative speech-in-noise performance data; as background noise levels increase, every participant (and patient) will have relatively increased difficulty understanding speech in background noise at some point. Some people may qualitatively rate their difficulties as more significant than others, even if their quantitative speech-in-noise test scores (and actual performance in real-world noise backgrounds) are equivalent. In other words, someone who self-reports difficulty understanding speech in noise, but has normal hearing thresholds and normal speech-in-noise test scores, may be functionally equivalent to others who do not report as much difficulty. Thus, a normal hearing person who self-reports difficulty understanding speech in noisy backgrounds may not necessarily have an abnormality or pathology (i.e., they may have normal hearing and speech-in-noise test scores), but could instead have different expectations regarding their performance across listening environments of varying difficulty (e.g., a one on one conversation in a co-worker's office vs. happy hour drinks with half the office staff at a busy restaurant). Such cases may potentially result in an opportunity for counseling of realistic speech-in-noise expectations and listening strategies, rather than a diagnosis of auditory dysfunction. The challenges of rehabilitation of deficits when patients do not meet the criteria for amplification were recently discussed by Kraus and White-Schwoch (2016), in their discussion of “Not-So-Hidden” Hearing Loss. This challenge of self-assessed perceptual difficulty directly parallels challenges related to the issue of tinnitus, as the self-assessed “bothersomeness” of tinnitus varies significantly from patient to patient, with no clear relationship to psychophysical parameters determined during pitch or level matching (for additional discussion, see Le Prell and Lobarinas, 2016).

There is an urgent need for validated, clinical tests that can be used to quantify patient self-report of difficulty understanding speech in noisy backgrounds. The ideal test will be sensitive to differences in performance within normal hearing listeners. For the WIN, a change of 3.5 dB-S/B (corresponding to a difference of approximately 4 words out of the 35 words presented) has been described as clinically meaningful (Wilson and McArdle, 2007). Many individual participants had changes of at least 4 words the day after noise exposure (Figure 5A), but not 1-week later (Figure 5E). The WIN should be considered for use in future studies not only based on the availability of the validated test as part of the NIH Toolbox (Zecker et al., 2013), but also based on the sensitivity of the test to acute, noise-induced changes in study participants. It may be the case that even greater sensitivity could be achieved in tests completed with higher background noise levels, or perhaps modifications (such as those of Liberman et al., 2016) that more appropriately reflect and reproduce the difficult listening environments found in real-world noisy and reverberative environments, such as restaurants, gymnasiums, bars, clubs, and other common venues with significant background noise.

The second part of this investigation was a prospective study measuring changes in audiologic function after new, acute recreational noise exposure. Audiometric, electrophysiological, and functional measures were monitored subsequent to noise exposure. There was no evidence that common recreational exposures resulted in permanent audiometric, functional, or electrophysiological deficits. Selective cochlear synaptopathy, resulting in an accompanying reduction in ABR Wave-I amplitude, has been clearly demonstrated in animals in association with noise exposures that induce a robust TTS the day after the noise exposure (for reviews, see Kujawa and Liberman, 2015; Liberman and Kujawa, 2017). The common, real-world recreational noise exposures that our participants experienced at concerts, multi-day music festivals, loud bars, etc. (see Figure 3), did not result in robust TTS the day after the exposure (most TTS < 15 dB, see Figure 8), nor did they result in decreases in AP amplitude (see Figure 7). Thus, they did not produce any evidence that would be interpreted as consistent with new, noise-induced cochlear synaptopathy following common, recreational noise exposure.

TTS was highly variable across individuals, which is a major challenge for studies such as these. The variability in TTS was consistent with that reported by others, as individual variability is significant after free-field exposures (Mills et al., 2001; Strasser et al., 2003) as well as controlled exposures delivered via personal music player devices (Le Prell et al., 2012, 2016; Kil et al., 2017). Across music player studies, a 100% noise dose (based on 29 CFR 1910.95) has resulted in highly variable TTS across participants, ranging from 0 dB to approximately 20 dB at 4 kHz, but with largely complete recovery the following day. Most, if not all, assessments of the effects of recreational noise have been completed immediately post exposure, with changes frequently being on the order of 8–10 dB as participants exit concerts (Opperman et al., 2006; Derebery et al., 2012; Ramakers et al., 2016) or clubs (Kramer et al., 2006); thus, the current data contribute further insight to the potential for changes in audiologic function the day after recreational exposure.

Our study found no statistically significant reduction in AP amplitude the day after exposure to common, loud, recreational events (see Figure 7). Although some participants had TTS exceeding typical test-retest of ±5 dB, there were no statistically significant relationships between changes in audiometric threshold sensitivity and noise dose (see Figure 4). There was a temporary statistically significant decrease in performance on the WIN test as a function of noise exposure in the overall analysis the day after the noise exposure (see Figure 5A), and for a small number of participants, the temporary deficits met the definition of clinically significant decrease in performance. However, due to a lack of a statistically significant decrease in either DPOAE amplitude (see Figure 6) or AP amplitude (see Figure 7) as a function of increasing noise exposure, it is not possible to directly attribute changes in performance on the WIN to specific OHC or synaptic injuries. It is possible to speculate that OHC injuries are more likely to underlie changes in performance on the WIN, based on the decreasing DPOAE amplitude at 6 kHz that was observed with increasing TTS (see Figure 8I), but these changes were limited to one frequency and it is not clear why 3 and 4 kHz failed to show similar noise-induced changes.

All of the evidence from animal models to date indicates that if noise-induced synaptopathy develops, it is immediate, and it is permanent. Thus, data from this prospective study showing temporary noise-related changes in performance on the WIN, in the absence of relationships between noise-exposure and changes in DPOAE and AP amplitude the day after noise exposure, cannot be interpreted as consistent with or otherwise suggesting synaptopathic damage in these human participants. In live human participants, cochlear synaptopathy cannot be directly measured, as synapse counts require *ex vivo* extraction of the temporal bone. The only direct evidence of synaptopathy in human cochlear tissues comes from Viana et al. (2015), who provided preliminary evidence of an age-related synaptopathy based on differences across five temporal bones. Those data are supplemented by Makary et al. (2011), who documented an age-related decrease in spiral ganglion survival which could be secondary to an age-related loss of their synaptic targets. Temporal bones may be a resource for new tissues, but unfortunately, noise history data are not always available for these tissues.

Human studies to date have generally relied on the amplitude of ABR Wave-I or the AP as an indirect proxy for potential synaptopathy (Stamper and Johnson, 2015a,b; Liberman et al., 2016; Bramhall et al., 2017; Prendergast et al., 2017; Spankovich et al., 2017; Fulbright et al., in press). ABR Wave-I amplitude has been highly correlated with synaptopathy in the animal studies thus far (Liberman and Kujawa, 2017); however, ABR measurements in anesthetized animals are much “cleaner” than ABR measurements in awake, resting humans.

At this time, there are no functional consequences that have been reliably associated with decreases in ABR Wave-I amplitude in normal hearing listeners. Bramhall et al. (2015) showed a statistically significant relationship between ABR Wave-I amplitude and performance on the QuickSin, but only in the presence of overt hearing loss; no statistically significant relationship was demonstrated between ABR Wave-I amplitude and performance on the QuickSin in participants with normal hearing. Data from rats showed that the functional deficits associated with decreases in ABR Wave-I amplitude were limited to the frequencies at which ABR Wave-I amplitude was decreased, and functional deficits were observed only in the most difficult listening condition (poorest signal to noise ratio) tested (Lobarinas et al., 2017). Although it is not clear how directly these results will translate to humans, it remains reasonable to hypothesize that speech-in-noise tests have the potential to reveal noise-induced deficits prior to the development of overt hearing loss in humans.

There are a variety of suggestions for other electrophysiological and psychophysical tools that might be considered for detection of hidden hearing loss in humans; various proposed metrics include the envelope following response (EFR) (Shaheen et al., 2015; Paul et al., 2017), middle ear muscle reflex (Valero et al., 2016), psychophysical manipulation of amplitude modulation in detection tasks (Paul et al., 2017), ABR Wave-V latency changes during forward masking (Mehraei et al., 2017), and binaural detection (Bernstein and Trahiotis, 2016). There are also suggestions to consider normalizing the amplitude of ABR Wave-I relative to the amplitude of ABR Wave-V (a measure of central response that does not appear to be affected by synaptopathy) (Verhulst et al., 2016), or relative to the amplitude of the summating potential (i.e., SP/AP ratio) (Liberman et al., 2016).

The argument that the SP/AP ratio is useful in revealing selective neural damage is based on the premise that SP is dominated by the OHC receptor potential (which is not expected to be affected by damage to the IHC/AN synapses), whereas AP is generated by the cochlear nerve. Early work by Durrant et al. (1998) attempted to resolve controversy over the relative contributions of the IHC and OHC populations to the SP; they concluded that while the OHCs made a significant contribution, the IHCs had a relatively greater contribution to the SP. Additional arguments that SP is appropriate for use normalizing AP are based on the observation that SP is more stable than AP after a variety of insults (for discussion see Liberman et al., 2016). However, the stability of SP may, in part, rely on the use of stimuli that are matched with respect to sensation level (i.e., the dB amount above individual threshold), as SP amplitude was constant across mice only when signal levels were equal sensation level (Sergeyenko et al., 2013). Furthermore, we point to data from Nam and Won (2004), who measured SP and AP after inducing TTS in human participants. They found that SP amplitude increased, but AP amplitude was unchanged, resulting in an increase in the SP/AP ratio. This finding parallels the increase in SP reported by Liberman et al. (2016) and, like Liberman et al. (2016), evidenced noise-induced changes in the SP/AP ratio to be driven by increased SP amplitude. If the SP is the measure relatively more affected by noise exposure, then the AP is essentially being normalized against a moving target, which seems counter-intuitive to the identification of selective neural deficits. Taken together, the noise-induced changes in SP and corresponding changes in SP/AP ratio (given that AP was unchanged) in those studies may be more appropriately interpreted as consistent with OHC based dysfunction, rather than synaptic neural dysfunction. Increasing OHC dysfunction as a function of increasing TTS was detected here (at least at 6 kHz), and noise-induced OHC dysfunction would be consistent with new work from Hoben et al. (2017) which importantly suggests that OHC loss or dysfunction may drive speech-in-noise deficits. Of note, the SP waveform is generally more difficult to resolve (Roland and Roth, 1997), and is highly variable across normal hearing listeners (Ferraro et al., 1994). In the current study, SP data were collected, as per the methods section, to permit calculation of SP/AP ratios following Liberman et al. (2016). Approximately 45% of the right and left ears had scorable SPs across the stimulus conditions. We initially included this ratio in the retrospective regression models, and found no statistically significant relationships detected for the subset of participants with reliable SPs. However, based on the above general concerns regarding the use of SP/AP ratios to identify selective neural damage, we did not assess potential changes in this ratio as a function of acute recreational noise exposure. Regardless, there was no relationship between AP amplitude and retrospective noise history (Figure 2), AP amplitude and acute recreational noise exposure (Figure 7), or change in AP amplitude and maximum observed TTS (Figure 8).

Other approaches that have been presented at recent scientific meetings include the normalization of ABR Wave-I amplitude for 4 kHz signals relative to ABR Wave-I amplitude for 1 kHz signals (Earl et al., 2017), and ABR Wave-I latency based comparisons instead of amplitude based comparisons (Skoe et al., 2017). As these different metrics and measures make their way through the peer-review process, it will hopefully become possible to begin to define the most informative strategies for those seeking evidence of hidden hearing loss in humans. If metrics selected for use in future studies include high level tone pips, some caution may be warranted with respect to interpretation of the frequency-specific effects; it is possible that high level tone bursts will activate relatively broader regions of the cochlea, perhaps even resembling the response to a click stimulus. The lack of agreed on metrics is clearly a major issue for translational human studies on hidden hearing loss (Le Prell and Lobarinas, 2016; Hickox et al., 2017; Kobel et al., 2017; Liberman and Kujawa, 2017).

## Summary and conclusions

The current investigation provided no evidence of noise-induced decreases in human AP amplitude in the retrospective analyses of noise exposure history, nor in the prospective analyses following common recreational noise exposure. The current data indicate that intra-participant changes in AP (ABR Wave-I) amplitude can be reliably monitored longitudinally; response waveforms were reliable and repeatable within individual participants, within and across sessions.

In animal models, the gold standard for identification of cochlear synaptopathy is the post-mortem counting of synaptic ribbons. Reductions in synapse count are highly correlated with the amplitude of Wave-I of the ABR (Sergeyenko et al., 2013). Liberman and Kujawa (2017) have therefore suggested that when DPOAE amplitude has returned to baseline (after noise exposure), or has not yet deteriorated (in the case of aging), the amplitude of ABR Wave-I is highly predictive of cochlear synaptopathy. In humans, there is a search for supra-threshold evoked potential metrics that will be sensitive to and specific for cochlear synaptopathy. The clinical (i.e., functional, “real-world”) relevance of reduced ABR Wave-I amplitude (AP amplitude) remains to be determined, despite much speculation. Even if a permanent noise-induced reduction of human ABR Wave-I amplitude is found following noise exposure in human participants, a meaningful, real-world functional effect must be identified in order for the ABR Wave-I amplitude reduction to serve as a clinically relevant finding in audiology. Here, the correlation analyses revealed a statistically significant relationship between noise dose/TWA and change in performance on the WIN, with statistically significant growth in deficits as TWA increased. For the majority of the participants, the individual noise-induced changes in WIN performance were small (1–3 word deficits at the test session the day after recreational noise exposure) but there were some participants with deficits of 4–6 words, which meets the criteria set by Wilson and McArdle (2007) for clinically significant change.

To be successful in the identification of noise-induced synaptopathic deficits in humans, it may ultimately be the case that future studies will need to include human populations exposed to noise insults that result in the magnitude of TTS minimally necessary to observe synaptopathic injury in animals. Such TTS changes appear to be unlikely to be produced from common recreational noise exposure, but are perhaps likely to be observed within military cohorts or safety officers, based on the data of Bramhall et al. (2017). Weapons training may provide more controlled access to noise-exposed participants, but enrollment in hearing conservation studies can influence the use of hearing protection devices (HPDs) that prevent the deficits of interest (see for example Le Prell et al., 2011). Regardless of the boundary at which risk begins, or the specific relationship between TTS and “hidden hearing loss,” it may ultimately prove difficult to identify a human cohort exposed to noise that is loud enough and long enough to cause neural damage, but leaves OHC function unaltered. This specific challenge was recently discussed in detail by Hickox et al. (2017), who point to the prevalence of mixed pathologies in human populations. A major remaining unknown is the extent to which repetition of noise exposure has the potential to result in a synaptopathic injury over time if smaller TTS changes are induced at each exposure (for additional discussion see Dobie and Humes, 2017; Murphy and Le Prell, 2017).

It is possible to imagine changes in conventional test batteries and/or metrics used for monitoring the effects of noise exposure if there were both compelling evidence of ABR Wave-I amplitude changes and accompanying functional deficits following noise exposure. Further research will be needed to more carefully assess the effects of noise exposures that have the potential to result in more severe TTS. Ethical practices for educating participants about the potential for auditory injury will need to be carefully considered, as per the recent commentary on TTS studies by Maison and Rauch (2017). Participants should be provided with HPDs if the investigator has reason to believe that the participant may be at risk for acoustic trauma resulting in permanent functional changes on threshold or suprathreshold measures of function. Such studies will also need to carefully assess OHC function and threshold sensitivity (including EHF threshold assessment) in order to systematically differentiate between OHC damage and potential neural synaptic damage, and document both overt and relatively more hidden supra-threshold hearing deficits.

## Ethics statement

This study was carried out in accordance with the recommendations of the Institutional Review Board at the University of Texas at Dallas with written informed consent from all subjects. All subjects gave written informed consent in accordance with the Declaration of Helsinki. The protocol was approved by the Institutional Review Board at the University of Texas at Dallas.

## Author contributions

SG: contributed to study design, data collection, data interpretation, and writing the manuscript. KW: contributed to study design, data collection, statistical analysis, data interpretation, and writing the manuscript. JB: contributed to study design, data collection, data interpretation, and reviewed the manuscript. CL: contributed to study design, statistical analysis, data interpretation, and writing the manuscript.

### Conflict of interest statement

The authors declare that the research was conducted in the absence of any commercial or financial relationships that could be construed as a potential conflict of interest. The authors alone are responsible for the content and writing of the paper. Support for this research was provided by the Emily and Phil Schepps Professorship in Hearing Science at the University of Texas at Dallas. Andrew Smith of Studio 6 Digital donated download codes for the “SPL Graph” smartphone app used by study participants.

## Abbreviations

ABR: Auditory brainstem response
AE: annual exposure
ASSR: Auditory Steady State Response
dB: decibel
dBA: A-weighted decibel
dB HL: decibel hearing level
dB S/B: decibel signal to babble ratio
dB SPL: decibel sound pressure level
DPOAE: distortion product otoacoustic emission
ECochG: Electrocochleography
EHF: extended high frequency
EL: exposure level
Hz: hertz
IHC: inner hair cell
kHz: kilohertz
L_Aeq8760_: A-weighted equivalent sound level 8760 h
NIOSH: National Institute on Occupational Safety and Health
NU-6: Northwestern University Auditory Test Number 6
OHC: outer hair cell
OSHA: Occupational Safety and Health Administration
PTS: permanent threshold shift
SRT: Speech Recognition Threshold
TTS: temporary threshold shift
WRS: Word Recognition Score
WIN: Words-in-Noise.
